# Photoacoustic transfection of DNA encoding GFP

**DOI:** 10.1038/s41598-018-37759-1

**Published:** 2019-02-22

**Authors:** Alexandre D. Silva, Carlos Serpa, Luis G. Arnaut

**Affiliations:** 0000 0000 9511 4342 grid.8051.c CQC, Department of Chemistry, University of Coimbra, Rua Larga, 3004-535 Coimbra, Portugal

## Abstract

Photoacoustic transfection consists in the use of photoacoustic waves, generated in the thermoelastic expansion of a confined material absorbing a short pulse of a laser, to produce temporary mechanical deformations of the cell membrane and facilitate the delivery of plasmid DNA into cells. We show that high stress gradients, produced when picosecond laser pulses with a fluence of 100 mJ/cm^2^ are absorbed by piezophotonic materials, enable transfection of a plasmid DNA encoding Green Fluorescent Protein (gWizGFP, 3.74 MDa) in COS-7 monkey fibroblast cells with an efficiency of 5% at 20 °C, in 10 minutes. We did not observe significant cytotoxicity under these conditions. Photoacoustic transfection is scalable, affordable, enables nuclear localization and the dosage is easily controlled by the laser parameters.

## Introduction

Gene therapy holds the promise of changing the quality of life of patients suffering from devastating genetic diseases such as hemophilia, Huntington’s Disease or cystic fibrosis. After decades of intensive research and hundreds of clinical trials, four gene therapies were recently approved: Glybera® (uniQure B. V.) for familial lipoprotein lipase deficiency, Imlygic® (Amgen) for melanoma, Strimvelis® (GlaxoSmithKline) for adenosine deaminase severe combined immunodeficiency and Luxturna® (Spark Therapeutics) for retinal dystrophy^1^. All these gene therapies rely on viral-vector gene transfer systems, which are the mainstream of gene transfer methods^2^. The main advantage of viral transduction is the high efficiency of foreign DNA introduction in cells, which *in vitro* may exceed 90%. However, viral vectors have a limited packing potential, raise concerns related to mutagenesis and immunogenicity, and the approved viral-vector gene therapies have a high cost^3^.

Affordable methods of transfection, such as the Ca-DNA co-precipitation, suspension and transport into cells by endocytosis^4^, are known for decades and can be very efficient *in vitro*^5^, but their use *in vivo* seems to be very limited. More recently, membrane-disrupting-based delivery methods are attracting much interest in view of their potential to deliver almost any cargo to any cell type^6^, although they still have limitations related to the balance between insufficient delivery and excessive cell damage, to scalability and to the inadequate understanding of cell recovery, resulting in inefficient protocols. Nevertheless, electroporation proved efficient for various cell types, reached commercial applications and is probably the nonviral approach to gene delivery *in vivo* with greatest impact to date^7^. Acoustic methods are another physical approach to transfection with the advantage of scalability, directionality and compatibility with repeated applications. Moreover, as other physical methods of transfection, acoustic methods avoid endocytosis and retention in lysosomes, inherent to many chemical-based gene delivery systems. However, the use of acoustic energy alone is associated with a low transfection efficiency compared with viral vectors and optimal chemical formulations^8,9^.

Acoustic methods can be divided in three main classes: conventional ultrasound (US), shock waves (including laser-induced stress waves, LISW) and photoacoustic (PA) waves. The acoustic waves of therapeutic (US frequencies ≈1 MHz) or diagnostic (US frequencies 1.5–15 MHz) ultrasound are characterized by compressional and rarefactional peaks of comparable amplitudes^10^. When such acoustic waves propagate in liquids or tissues, they induce cavitation which, for critical acoustic energies, implode and produce shock waves. It is believed that such shock waves, and the projections associated with them, are responsible for increasing cell membrane permeability to DNA and, for the same reasons, to cell death. The threshold for cavitation can be lowered with the introduction of microbubbles in the media of interest. Nevertheless, it is very difficult to control the exposure of cells to cavitation and find the best balance between transfection efficiency and cell viability.

This work explores the use of PA waves in gene transfection. PA waves are predominantly compressive pressure waves generated when radiationless processes rapidly convert the energy of a short laser pulse into heat in an absorbing material. PA waves are launched in the material if the laser pulse duration (*τ*_L_) is shorter than the stress relaxation time (*τ*_s_), where *τ*_s_ = 1/(*μ*_a_*c*_s_) (*c*_s_ is the speed of sound and *μ*_a_ is the linear absorption coefficient), i.e., if the laser pulse width is of a few nanoseconds or of picoseconds. We describe PA waves generated by the absorption of short laser pulses in piezophotonic materials, and the transfection of a plasmid DNA encoding Green Fluorescent Protein (gWizGFP, 3.74 MDa) in COS-7 monkey fibroblast cells. Obara and co-workers described before the use of LISW to deliver plasmid DNA into cells^11–16^. Although LISW and PA waves probably share the same gene transfection mechanism – transient disruption of the plasma membrane by mechanical deformation^12,17^ – stress waves generated by laser ablation of a target are of a fundamentally different nature than photoacoustic waves generated by thermoelastic expansion. Laser ablation uses high laser fluences (≈1 J/cm^2^) that generate high peak pressures (≈50 MPa) with relatively long rise times (≈160 ns)^14^. These conditions are destructive of the target and only very few laser pulses (e.g. <40) can be used^18^, leading to transfection efficiencies often lower than 1% at 37 °C^14^. The thermoelastic expansions generated by laser pulses with relatively low laser fluences (<100 mJ/cm^2^) lead to very reproducible high frequency ultrasound waves that can be used thousand times without toxicity^17,19^. Their safety was recently emphasized in aesthetic medicine^20^. We take advantage of the safety profile of high frequency US waves generated by photoacoustic phenomena to expose COS-7 cells to thousands of laser pulses without cytotoxicity, and increase the transfection efficiency.

## Results

### Characterization of photoacoustic waves

We generated PA waves with a dye incorporated in a thin (≤80 µm) polymer film, such that the absorbance of the film is between 1.2 and 1.4 at 532 nm, and the film is confined between a rigid glass window and a back mirror. The dye in the film converts the radiative energy of a 532 nm laser pulse in thermal energy within the duration of the laser pulse. The subsequent thermoelastic expansion is converted into a strong and sudden pressure increase by the effect of confinement by rigid boundaries^17^. This pressure pulse travels as an US wave across the mirror and propagates in the medium in contact with the mirror.

**Figure 1:**
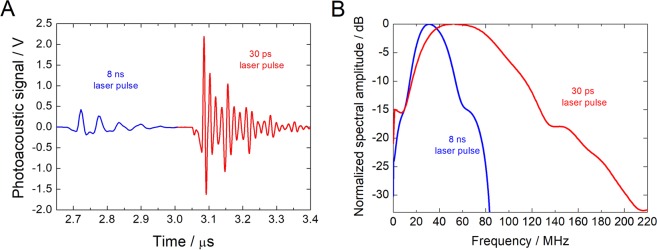
(**A**) Representative PA waves measured by a 100 MHz contact transducer produced by the piezophotonic material upon 100 mJ/cm^2^ pulsed laser excitation at 532 nm, with *τ*_L_ = 8 ns (blue) and *τ*_L_ = 30 ps (red). (**B**) FFT of the PA waves collected with 225 MHz transducer.

Figure 1A presents typical PA waves as seen by a 100 MHz contact transducer coupled to the mirror of the piezophotonic device. Figure 1B presents the Fast Fourier Transform (FFT) of PA waves obtained under the same conditions with a 225 MHz contact transducer. The response of this transducer is within −6 dB (i.e., power reduction by a factor of 4) of its maximum sensitivity at 225 MHz in the frequency range between 88 and 318 MHz. Hence, the contribution of frequencies below 100 MHz to the observed signal tends to be underestimated. Nevertheless, it is possible to see that 30 ps laser pulse leads to ultrasound with higher components of very high frequencies (i.e., above 100 MHz). Although the 100 MHz transducer may introduce some bias in the detection of the ultrasound generated by the nanosecond laser, Fig. 1A shows that the ultrasound waves generated by the picosecond laser are 5x more intense than the waves generated by the nanosecond laser. This difference is likely to reflect the higher optical power density, *I*_L_, of the picosecond laser at the laser fluence of 100 mJ/cm^2^ (*I*_L_ = 0.0125 GW/cm^2^ and 3.33 GW/cm^2^ for *τ*_L_ = 8 ns and 30 ps FWHM pulses, respectively).

**Figure 2:**
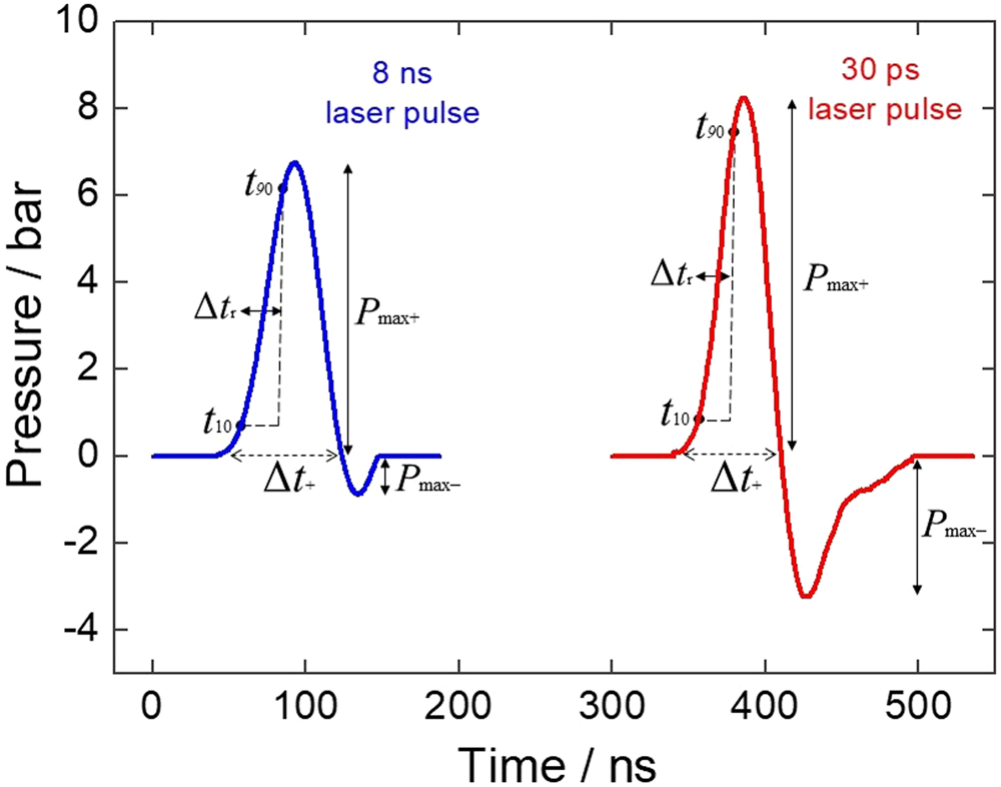
Pressure waves produced by the piezophotonic material as a result of absorption of a 100 mJ/cm^2^ laser pulse at 532 nm and measured by a 20 MHz needle hydrophone. Blue (left) and red (right) lines correspond to the pressure waves obtained with irradiation by a nanoseconds laser and picoseconds laser, respectively.

Figure 2 presents the absolute pressure waves measured with the 20 MHz needle hydrophone. The pressures were calculated using the sensitivity factor of the hydrophone, which is calibrated only for the 1–20 MHz range. Hence, the center frequency of the broadband ultrasound generated by the picosecond laser may be outside this frequency range and the absolute pressure is likely to be underestimated. Table 1 presents the pressure parameters of the PA waves represented in Fig. 2, namely the peak compressional and rarefactional pressures (*P*_max+_ and *P*_max−_), the duration of the compressional pressure (∆*t*_+_), and the rise time of the first positive half-cycle (∆*t*_r_) defined as the time from 10% to 90% of the peak pressure. Additional properties of ultrasound waves often related to cell permeabilization to large molecules^16,21^ are the stress gradient, calculated as *S* = *P*_max+_/∆*t*_r_, and the impulse *I*_p_, defined as the pressure integrated over the time of the compressional wave ∆*t*_+_.

**Table 1:** Properties of PA waves.

Microphone	*τ*_L_ ns	*P*_max+_ bar	*P*_max−_ bar	∆*t*_+_ ns	∆*t*_r_ ns	*S* bar/ns	*I*_*p*_ μbar s
20 MHz	8	6.8	0.9	75	27	0.25	2.6
	0.03	8.3	3.2	60	23	0.36	2.7

### Cell viability

**Figure 3:**
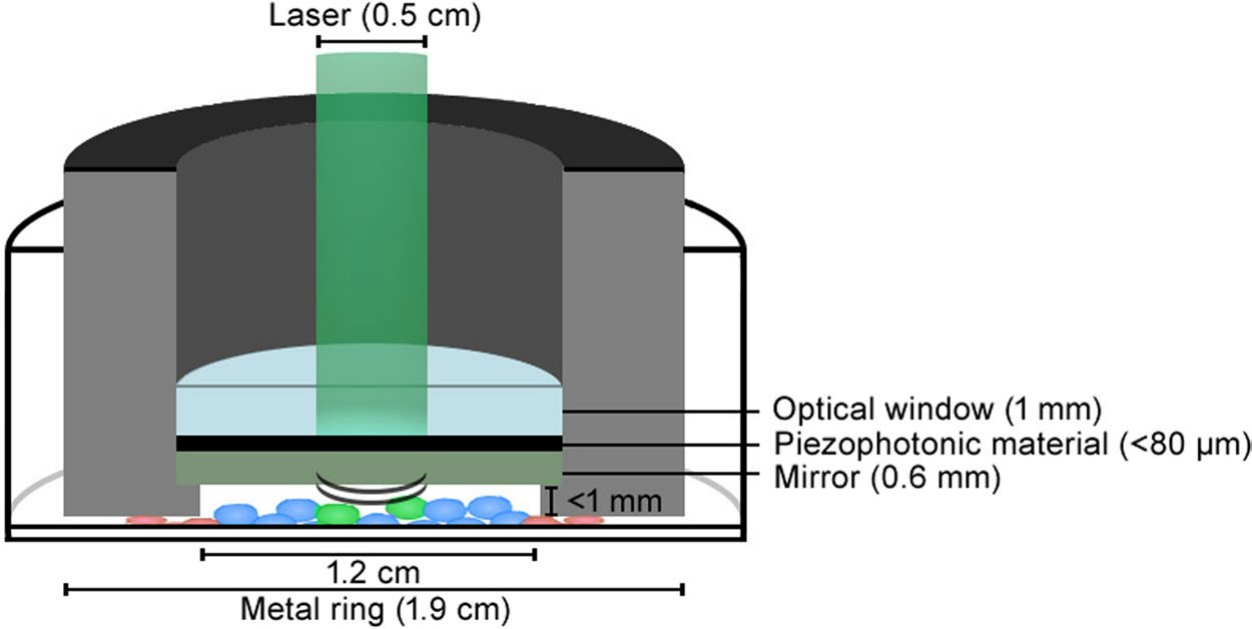
Experimental setup for generation of PA waves.

The set-up employed to expose the cells to PA waves is shown in Fig. 3. When 532 nm laser pulses are directed through a glass window to a light-to-pressure conversion material (i.e., a “piezophotonic” polymer film), more than 94% of the photons in the laser pulse are absorbed by the film and the remaining 6% are reflected by the mirror and make a second passage though the film. PA waves are generated in the confined film and travel through the mirror to the cell culture. The mirror is positioned ca. 1 mm from the monolayer of cells. This distance is determined by the thickness of a metal ring where the mirror seats. The cell culture medium provides the acoustic coupling between the mirror and the cells. The metal ring has an outer diameter practically coincident with that of the cell culture dish (ca. 19 mm) but the laser beam diameter is only 5 mm. The area of the ultrasound source is 1/5 of the total area where cells were seeded. The metal ring seats on cells growing in the border of the well. The mechanisms of cell death were investigated using the Hoechst 33342 probe to identify apoptotic cells and Propidium Iodide (PI) to stain dead cells (possibly necrotic when stained shortly after the insult). The Supplementary Information shows that 4 h after exposure to the PA waves some cells in the center of the dish incubated with Hoechst 33342 probe exhibit blue fluorescence (Supplementary Fig. S1), but when incubated with PI they do not exhibit red fluorescence. This suggests that some apoptotic cells may be present in the center of the dish that was exposed to PA waves. On the other hand, incubation with PI leads to substantial red fluorescence from the borders of the dish (Supplementary Fig. S2), indicating that the cells in contact with the metal ring are dead.

**Figure 4:**
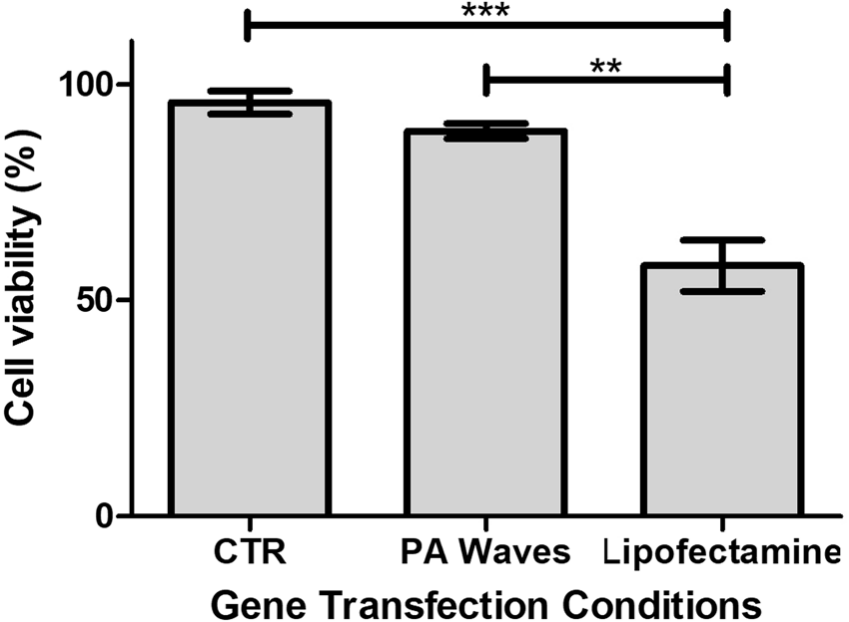
Cell viability of COS-7 cells for different transfection conditions. PA waves were generated for 10 min with 30 ps laser pulses at the fluence of 100 mJ/cm^2^. Cell viability was evaluated 24 h after the exposure to PA waves or the addition of Lipofectamine. **p = 0.0072, ***p = 0.0002.

Figure 4 presents the viability of cells exposed to Lipofectamine® 2000 (4 μL per well, 4 h of incubation) or PA waves (30 ps laser fluence of 100 mJ/cm^2^ for 10 min) assessed with the alamarBlue® assay. Lipofectamine was used as positive control because it is widely accepted as “gold-standard” for the safe delivery of exogenous DNA or RNA into cells^22^. However, Lipofectamine reduces cell viability by more than 40% relative to control (*p* = 0.0002). On the other hand, the viability of cells exposed to PA waves in the most severe conditions studied in this work (30 ps laser at a fluence of 100 mJ/cm^2^, or *I*_L_ = 3.33 GW/cm^2^, for 10 min or 6,000 laser pulses) is not statistically significantly different from control under exactly the same experimental design but without light (*p* = 0.1450). The laser fluence employed in this study is limited by the maximum energy per pulse of the picosecond laser at 532 nm (30 mJ), and the exposure time is limited both by the fatigue of the piezophotonic material and by the time the cells must remain outside the laminar flow cabinet.

### Transfection with Lipofectamine^®^

**Figure 5:**
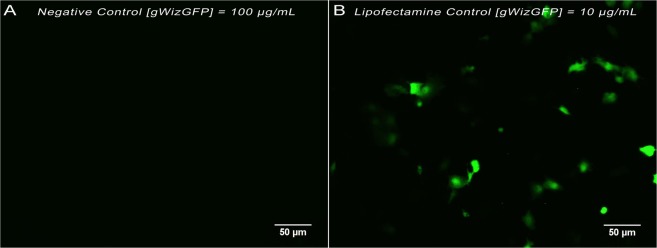
Representative images of GFP fluorescence by the  COS-7 cell line 24 hours after transfection with plasmid gWizGFP. The area represents an average of 250 ± 50 cells. (**A**) Negative control; [gWizGFP] = 100 μg/mL. (**B**) Lipofectamine (4 µL in 300 µL of culture medium); [gWizGFP] = 10 μg/mL.

Negative control, performed only in presence of gWizGFP plasmid, showed no transfected cells (Fig. 5A), confirming that such high molecular weight molecules do not cross the cell membrane without an intracellular delivery method. Figure 5B shows GFP expression by the  COS-7 cell line 24 hours after transfection with Lipofectamine® 2000. This is a well-known and efficient transfection reagent and was used in this work as a positive control. In the conditions of Fig. 5B, the concentration of Lipofectamine® 2000 present is responsible for more than 40% reduction in cell viability.

### Photoacoustic transfection

**Figure 6:**
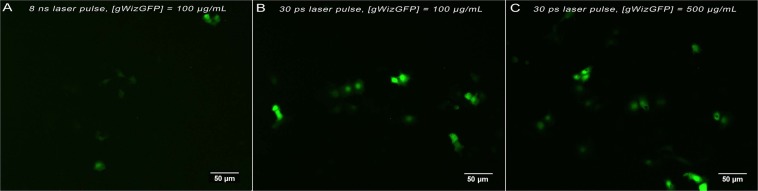
Representative images of GFP fluorescence from COS-7 cells 24 hours after transfection of the gWizGFP plasmid with 10 min exposure to PA waves generated with a 100 mJ/cm^2^ laser fluence. The area represents an average of 250 ± 50 cells. (**A**) [gWizGFP] = 100 μg/mL, 8 ns laser pulses. (**B**) [gWizGFP] = 100 μg/mL, 30 ps laser pulses. (**C**) [gWizGFP] = 500 μg/mL, 30 ps laser pulses.

Photoacoustic transfection of COS-7 cells with gWizGFP plasmid was first investigated semi-quantitatively to evaluate the various factors affecting the efficiency of transfection and to select the conditions for quantitative experiments. The following factors were evaluated: (i) exposure times of 3, 5 and 10 min (Supplementary Figs S3, S4 and S5); (ii) laser fluences of 33, 66 and 100 mJ/cm^2^ (Supplementary Figs S6, S7 and S8); (iii) plasmid concentrations of 100, 250 and 500 µg/mL (Supplementary Figs S9, S10 and S11) (iv) 8 ns and 30 ps laser pulses using plasmid concentrations of 100 µg/mL (Figs 6 and S12). For each experiment, series of images with the same size were collected from the area of the well exposed to PA waves and the three images with higher GFP fluorescence were selected for analysis. The number of cells transfected in each selected image was counted and related to the factor changed. Supplementary Figs S13 to S16 show that the transfection efficiency: (i) increases with the exposure time, (ii) increases with the concentration of the plasmid, (iii) increases with the laser fluence, (iv) is systematically higher for 30 ps pulses than for 8 ns pulses. Figure 6 shows representative images of GFP fluorescence 24 h after 10 min exposure of the cells to PA waves generated by the absorption of 100 mJ/cm^2^ laser pulses by piezophotonic materials, using pulse durations of 8 ns or 30 ps and different plasmid concentrations. These results show that pressure waves permeabilize cell membranes and promote transfection, which is consistent with reports on LISW^23^.

### Flow cytometry

**Figure 7:**
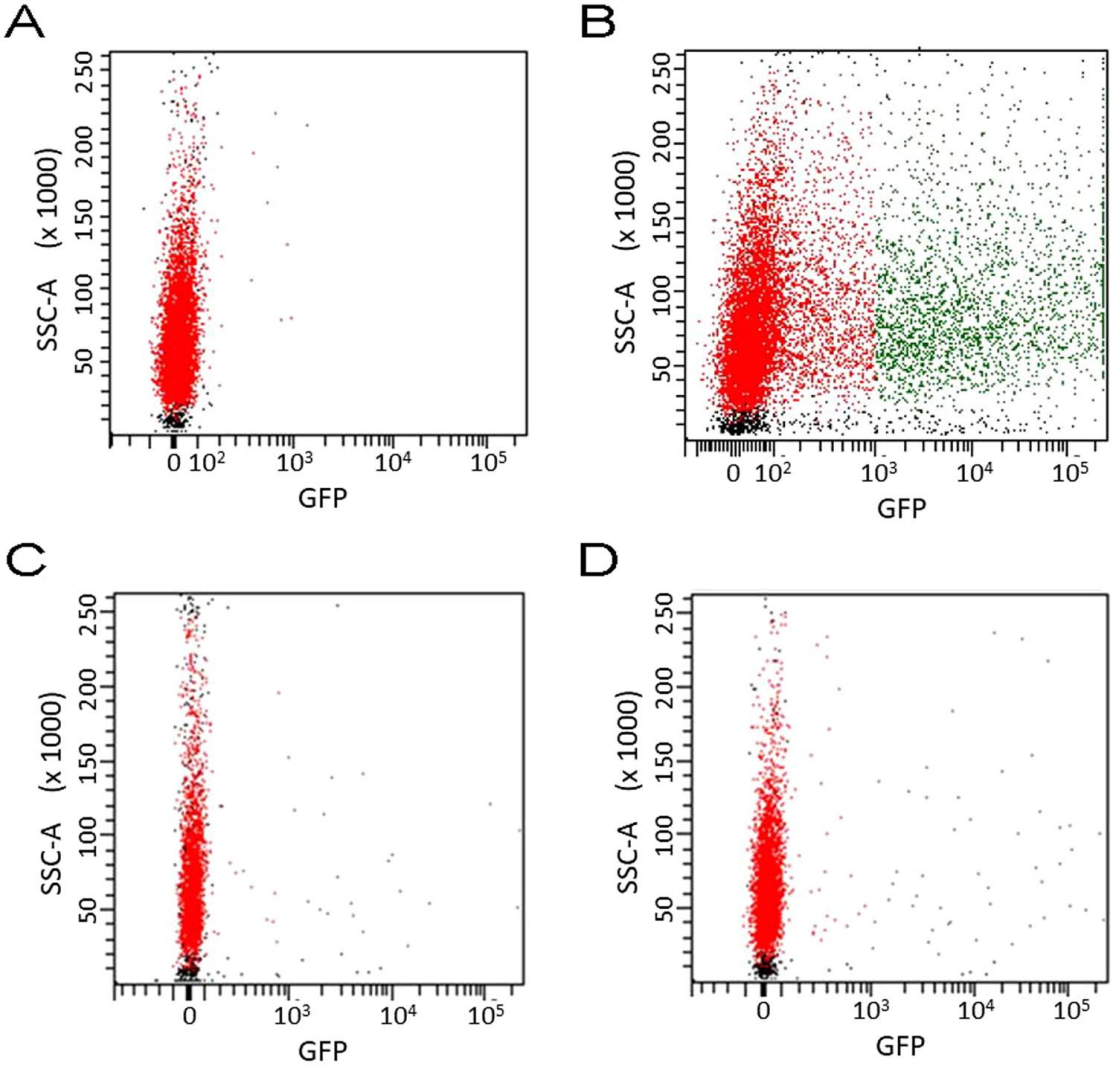
GFP expression (green dots) and cells side-scattered light (SSC), 24 h after transfection. (**A**) Control with no transfection method and incubation with [gWizGFP] = 100 μg/mL. (**B**) Transfection with Lipofectamine® 2000 using an incubation time of 4 h. (**C**) Transfection with PA waves for 10 min using 30 ps laser pulses with a 100 mJ/cm^2^ fluence and [gWizGFP] = 100 μg/mL. (**D**) Same as B but using [gWizGFP] = 500 μg/mL.

Flow cytometry using FACS (Fluorescence Activated Cell Sorting) was used to quantify the efficiency of transfection with Lipofectamine® 2000 using the most promising laser parameters and plasmid concentrations. Figure 7 shows the GFP expression in COS-7 cells 24 h after transfection with Lipofectamine® 2000 (incubation time of 4 h) or with the PA waves generated with a 30 ps laser (exposure time of 10 min). Two concentrations of plasmid were employed in the transfection with PA waves. The control (Fig. 7A), i.e., transfection without active delivery methods, was used to set the transfection threshold at 10^3^ in the GFP expression scale. Under our experimental conditions, Lipofectamine® 2000 transfected 23% of the cells (Fig. 7B). Transfection with PA waves (Fig. 7C,D) is clearly less efficient, reaching 0.6% and 0.9% of the cells for [gWizGFP] = 100 μg/mL or 500 μg/mL, respectively. However, it must be realized that the incubation with Lipofectamine® 2000 for 4 h affects all the cells evaluated, whereas the PA waves only affect 1/5 of the cell monolayer, due to the geometry of the device illustrated in Fig. 3. All the cells in the monolayer were detached and analyzed by flow cytometry, but only 1/5 of them were exposed to the PA waves. Hence, making this geometric correction for the actual exposure to the PA waves, the transfection efficiency with PA waves is 3.3% and 5.0% for [gWizGFP] = 100 μg/mL or 500 μg/mL, respectively. The transfection with Lipofectamine® 2000 was performed at 37 °C in the laminar flow cabinet, but the transfection with PA waves was performed at 20 °C outside the laminar flow cabinet. It is known that transfection efficiency is strongly dependent on the temperature^12^. Thus, the transfection efficiency of 5% with PA waves at 20 °C must be regarded as a conservative estimate of the transfection enabled by PA waves.

## Discussion

Efficient piezophotonic materials must have high linear absorption coefficients, ultrafast radiationless transitions and high thermal expansions^24^. The piezophotonic material used in this work contains MnTPP that strongly absorbs light at 532 nm and decays non-radiatively to the ground state with a lifetime shorter than 150 ps^25,26^. This ultrafast heat release produces a thermoelastic expansion of the polystyrene matrix that is frustrated by the rigid confinement of the piezophotonic material, leading to a fast pressure increase that launches an intense US pulse^27^. The peak compressional pressure (*P*_max+_) of PA wave is proportional to the energy absorbed below the ablation threshold, and inversely proportional to the thickness of the material for the same amount of energy absorbed^28,29^. The thin (<80 µm thickness) and strongly absorbing (*A*_532_ = 1.2 to 1.4) films used in this work have an optical penetration depth of *δ* = 1/*μ*_a_ ≈ 65 µm. This is a case of volume absorption, where the heat is deposited in the whole illumination volume, and the maximum temperature elevation is reached at the end of the laser pulse^30^. Considering that damage to the material is only related to the maximum temperature elevation, the laser pulse duration does not significantly change the damage threshold. The observation of *P*_max+_ ≈ 7 bar for each 8 ns laser pulse over thousands of laser pulses confirms the generation intense US waves at laser fluences below the ablation threshold of polystyrene. The higher *P*_max+_ obtained with the 30 ps laser agrees with the expected dependence on the laser peak intensity^31^, and the reproducibility of the corresponding PA waves for at least 10 min at 10 Hz shows that the ablation threshold was not attained although the optical power density reached 3.33 GW/cm^2^, in accordance to other systems^32^.

An interesting property of PA waves that obey the condition *A* = *μ*_a_*c*_s_*τ*_L_ ≫ 1 (*c*_s_ = 2400 m/s in polystyrene^33^), is that the acoustic pressure is proportional to the time derivative of the laser pulse, meaning that the center frequency (*f*  ) and bandwidth of the generated US is mainly determined by the incident laser pulse^31,34,35^. The nanosecond laser pulse is close to meet this condition but the picosecond laser leads to *A* ≪ 1. Figure 1 shows that the PA waves produced in this work contain a broad band of frequencies and that the frequency spectrum of the PA wave generated with the picosecond laser extends to frequencies higher than 100 MHz, but not to the GHz frequencies that could be attained if *A*»1 was fulfilled.

The low *P*_max−_ and the high frequency components of the PA waves used in this work place their mechanical index below the onset of cavitation, *MI* = *P*_max−_/√*f* < 0.7, where the pressure is in MPa and the frequency in MHz^36^. This contrasts with sonophoresis (*f* < 1 MHz) where the cavitation of microbubbles is responsible for transfection^37^ or dermal delivery^38^. On the other hand, the high frequencies and high *P*_max+_ of PA waves lead to high stress gradients and high impulses that enable less common mechanisms of interaction between US and cells. GHz frequency acoustic waves have been shown to interact with cell membranes and contribute to membrane deformation^39^. Reversible poration of the cell membrane with hypersound was shown to transfect plasmid DNA encoding GFP to HeLa cells^39^. A limitation of piezoelectric generation of such hypersound is the small size of the piezoelectric resonator. The active area of the piezophotonic material used in this work was 0.20 cm^2^, but the only limitation to use larger areas is the pulse energy of the laser. The scalability of photoacoustic transfection is simple and affordable.

The mechanism of interaction of the stress gradient of high-frequency, high-intensity US with cells is comparable with the application of hydrostatic pressure to a cell through a pipette. It was shown that the width of epithelial cells is elastically deformed by 2 µm when a 0.1 MPa pressure is exerted on the cells^40^. This mechanism can also be related with the compression and shear forces experienced by cells as they are deformed to pass through a constriction 30–80% smaller than the cell diameter in microfluidic platforms^41^. As the cells are squeezed, transient holes appear in the cell membrane and facilitate the diffuse delivery of a large variety of materials^41^. We have observed transient changes in the fluorescence anisotropy of diphenylhexatriene (DPH) incorporated in dipalmitoylphosphatidylcholine (DPPC/DPH vesicles) when exposed to PA waves generated by 8 ns laser pulses^19,42^. Such changes suggest that the radiation pressure exerted by intense and high frequency PA waves can change the properties of membranes. Evidence for transient membrane disruption by shear force has also been presented for the intracellular delivery of plasmid DNA with LISW^14^.

A major concern in gene therapy is the toxicity of the gene transfer methods. Figure 4 shows that, for the doses employed in our studies, the PA waves do not show cytotoxicity. The photoacoustic transfection efficiency obtained in this work is not limited by toxicity. This is not the case of Lipofectamine® 2000. This synthetic cationic liposome formulation forms complexes with nucleic acid molecules, allowing them to overcome the electrostatic repulsion of the cell membrane and to be taken up by the cell^43^. We used these lipoplexes as positive control, to compare photoacoustic transfections with an established method of *in vitro* transfection, but their 23% transfection efficiency comes with the cost of 45% cell death. The absence of toxicity with 1 MPa PA waves is not surprising in view of 100% viability reported by Doukas and coworkers after one single shock wave of 32 MPa^21^ and the ca. 90% viability reported by Obara and co-workers after 20 LISW of 50 MPa^14^. On the other hand, the plasmid concentrations required for efficient transfection with 1 MPa PA waves are one order of magnitude higher than those typically used in transfection with Lipofectamine® 2000.

Transfection efficiency was assessed 24 h post exposure to PA waves to allow for a direct comparison with the transfection efficiencies reported in the literature using other methods. However, it should be emphasized that at latter times the cells continue to produce GFP and that fluorescence intensity increases over time. The transfection efficiency in the monolayer exposed to 10 min of PA waves generated with a picosecond laser was 5.0% for plasmid vector concentrations of 0.5 mg/mL, respectively. This can be compared with the transfection efficiency of 2.3% for the same plasmid vector concentration obtained after one LISW of 50 MPa at 37 °C, which is associated with a survival rate of 87.5% relative to control cells^12^. Interestingly, the transfection efficiency increases to 5.8% but the survival rate drops to 80.3% when the exposure to LISW is made at 43 °C^12^. In addition to the difference in temperatures (20 °C vs. 37 °C or 43 °C), there is also a difference in the size of the plasmid vectors (5757 bps for gWizGFP vs. 4731 bps for pEGFP-C1), both biasing the comparison in favor of LISW. Clearly, exposure to 6,000 PA waves of 1 MPa described in this work is safer than exposure to a single 50 MPa LISW, and leads to higher transfection efficiencies. The much higher impulses offered by the large laser fluences (>1 J/cm^2^) employed in the generation of LISW do not seem to offer an advantage for transfection and are associated with cytotoxicity. On the other hand, the short durations of the PA waves, especially with picosecond lasers, generate very high frequencies that favor the generation of high stress gradients at modest peak pressures. This makes the cytotoxicity negligible even when the cells are exposed to thousands of PA waves, and allows for a prolonged exposure of the cells to PA waves, eventually leading to higher transfection efficiencies. This prolonged exposure is enabled by the fact that thermoelastic expansion at 100 mJ/cm^2^ laser fluences does not damage the piezophotonic materials, as opposed to ablation at 1 J/cm^2^. The low laser fluence employed to generate PA waves is compatible with the use of fiber optics and endoscopic methods to minimize the invasiveness of transfection in a clinical setting.

The transfection of a plasmid DNA in COS-7 cells with 5% efficiency using a laser fluence of 100 mJ/cm^2^ was made possible by the development of piezophotonic materials with high light-to-pressure conversion efficiencies^17^. These materials were developed to convert nanosecond laser pulses into PA waves capable of permeabilizing the *stratum corneum* and enhance transdermal drug delivery^19^. When such materials are employed with picosecond laser pulses, they should generate broadband PA waves with frequencies extending to GHz. However, the bandwidth of the US generated in this work is not determined by the incident laser pulse and we assign this limitation to the use of materials with linear absorption coefficients lower than required by the condition *A*»1. The appropriate design of piezophotonic materials to efficiently respond to picoseconds lasers (e.g., a material with *μ*_a_ > 2000 cm^−1^, a high thermal expansion coefficient and very short excited state lifetimes) may lead to intense, broadband US pulses with GHz frequencies that produce pressure gradients capable of substantially increase the transfection efficiency without added toxicity.

In summary, photoacoustic transfection is scalable, affordable, induces a minimal cell perturbation, enables nuclear localization and the dosage is simply controlled by the laser parameters. These, together with the ability to deliver materials with diverse chemical and physical properties, are the ideal features of next-generation intracellular delivery systems^6^. Although in this work we did not attempt to deliver diverse materials, previous studies using PA waves to deliver materials to, or through, the skin have shown that PA waves increase the flux of small molecules (e.g., H_2_O), large molecules (redaporfin), proteins (e.g., GFP) and large biopolymers (e.g., 800 kDa hyaluronic acid)^17,19^. The 5% efficiency demonstrated for transfection of the 3.74 MDa gWizGFP plasmid at 20 °C exposed to 10 min of PA waves generated with 100 mJ/cm^2^ laser pulses without cytotoxicity, may be improved with the use of piezophotonic materials capable of generating PA waves with the bandwidth the picosecond laser pulses, that will offer extraordinary opportunities for gene therapy.

## Materials and Methods

### Photoacoustic (PA) Wave measurements

The light-to-pressure transducer used in this work consisted of manganese (III) chloride 5, 10, 15, 20-tetraphenylporphyrinate (MnTPP) dye homogeneously incorporated in polystyrene films with thickness between 70 and 80 µm and absorbances between 1.2 and 1.4 at 532 nm, measured with a Cary 5000 Series UV-Vis-NIR Spectrophotometer (Agilent Technologies). In the photoacoustic measurements, this material was confined between a 1 cm thick quartz window and a 1 cm dielectric mirror to optimize the generation of ultrasonic pressure waves. Two types of measurements were performed, either using very high frequency (100 and 225 MHz) contact transducers from Panametrics/Olympus (models V2012 and V2113), or a 20 MHz needle hydrophone from Force Technologies (model MH28). The contact transducers allowed for the investigation of very high frequencies present in photoacoustic waves, whereas the needle hydrophone, calibrated in the 1 to 20 MHz range, measured absolute pressures. The measurements of the PA waves with the contact transducer used the front-face irradiation design of photoacoustic calorimetry^28^, with all the parts acoustically coupled with silicone. The measurements with the hydrophone were made in a water pool at 20 °C, with the piezophotonic film confined between a glass window 1 mm thick and a back aluminum mirror on a 0.6 mm thick plastic support. These light-to-pressure transducers have been named piezophotonic (light-to-pressure transducer) materials. The hydrophone was placed within 2 mm of the mirror. The pressure waves were calibrated on the basis of a sensitivity of 16.708 mV/bar provided by the manufacturer for the 1 to 20 MHz range. Photoexcitation of the dye in the polystyrene film employed the second harmonic (532 nm) of nanosecond (Spectra Physics Quanta Ray GCR-130, 8 ns pulse FWHM, 10 Hz) or picosecond (EKSPLA PL 2143 A, 30 ps pulse FWHM, 10 Hz) Nd:YAG lasers. The PA waves were registered with a DPO7254 Tektronix digital oscilloscope (2.5 GHz bandwidth). It has been reported in the literature that for large laser fluences, degradation of the laser target and decoupling of the various components of light-to-pressure transducers are visually observable. We verified that the PA waves remained reproducible after thousands of laser pulses, and that the piezophotonic material did not present signals of damage. Nevertheless, the piezophotonic materials used in this work were regularly replaced.

### Transfection *in vitro*

Monolayers of immortalized COS-7 monkey fibroblast cell line (ATCC) were cultured in Dulbecco’s Modified Eagle’s Medium (DMEM) supplemented with 10% heat-inactivated fetal bovine serum (Gibco) and 1% penicillin and streptomycin (Invitrogen), in humidified atmosphere with 5% CO_2_ at 37 °C. MilliQ water was deionized with a Millipore Milli-Q water purification system.

Transfection of the plasmid DNA encoding Green Fluorescent Protein (GFP) gWizGFP (3.74 MDa, Aldevron) with Lipofectamine® 2000 (Invitrogen™) was used as a positive control^43^. Plasmid DNA and Lipofectamine lipoplexes were prepared in growth medium (DMEM), without serum or antibiotics, by addition of Lipofectamine 2000 (4 μL per well) with the plasmid DNA at a concentration of 10 μg/mL. After 20 minutes of preparation, the lipoplexes were added to COS-7 cells adhered to the well surface of 12-well plates forming a monolayer with confluence levels close to 80–90%, to a final volume of 300 μL. After four hours of incubation, 1700 μL of culture medium supplemented with serum and antibiotic were added to the cell culture. The lipoplexes remain incubated with the cells for additional 20 h, after this dilution by a factor of 6.7^43,44^.

Transfection of the same plasmid with PA waves employed the set-up presented in Fig. 3. First, COS-7 cells were seeded in 12-well plates at a density of 30.000 cells/well in 2 mL of growth medium. Twenty hours later, the culture medium was removed and 300 μL of plasmid solution in culture medium was added without serum and antibiotics. Then, the piezophotonic material was immersed in the culture medium and brought to close proximity of the cell monolayer but without physical contact. The laser was turned on for different times up to 10 min (i.e., up to 6,000 laser pulses), exposing the cells to PA waves. Plasmid concentrations of 100 μg/mL, 250 μg/mL and 500 μg/mL were tested. The maximum laser fluence employed was 100 mJ/cm^2^, both for nanosecond and picosecond lasers. This limit was imposed by the maximum pulse energy available for the picosecond laser. The use of the set-up in Fig. 3 required removing the cells from the laminar flow cabinet (Thermo Scientific, MSCAdvantage), and for a total of 90 min they were kept in normal atmosphere at 20 °C. Cell viability controls were kept in the same conditions both the experiments with Lipofectamine® 2000 and for PA waves, i.e., independent controls were made for these two types of experiments.

### Cell Viability and Transfection Efficiency

Cell viability was measured using the Alamar Blue® assays. In this assay 600 μL of medium culture containing 10% Resazurin was added to the cells 24 hours after the exposure to the appropriate transfection method (PA waves or Lipoplexes)^45^, and fluorescence was analyzed with a Microplate Reader (Synergy HT™ from BioTek®) exciting at 530 nm and detecting the emission at 590 nm. The cell viability was obtained using the expression:

$$Cell\,Viability\,( \% )=\frac{Fl\,590\,nm\,treated\,cells}{Fl\,590\,nm\,control\,untreated\,cells}\times 100$$

The cell death at several time-points after the exposure to the PA waves was investigated with the fluorescent DNA-staining probes Hoechst 33342 and Propidium Iodide (PI) using an inverted microscope (Olympus CKX41SF-5) coupled to a fluorescence system (Olympus U-RFLT50) and following the indications of the supplier (Invitrogen).

Transfection efficiency was evaluated in terms of GFP expression in the cells 24 h after exposing cells to lipoplexes or to PA waves. In the first exploratory studies GFP expression was evaluated using the microscope described above using the excitation filter BP460–490 nm and the fluorescence detection filter BA520IF. Once the parameters for efficient transfection were established, the transfection efficiency (i.e., the percentage of cells expressing GFP) was obtained by flow cytometry. The flow cytometer (BD FACSCanto™ II, BD Biosciences) was set to collect data on 5000 cells with excitation at 488 nm and detection of green fluorescence. FACSDiva version 6.1.3 software was used to analyze experimental data. The same number of cells was evaluated for each sample, correcting for any difference in cell density.

### Statistical Analysis

All data are expressed as mean values or expressed as a percentage relative to control ± SEM. Statistical differences between groups were determined using Student’s t-test for independent samples and GraphPad Prism 5 software. Statistical significance was defined as *p* <0.05. Alpha value 0.05 and two-tailed calculations were used where applicable.

## Supplementary information

Supplementary Information Photoacoustic transfection of DNA encoding GFP

## Data Availability

Data described in this study will be available from the corresponding authors upon request.
