# Supplementary material for: Photoacoustic transfection of DNA encoding GFP
Source: Sci Rep. 2019 Feb 22;9:2553. doi: 10.1038/s41598-018-37759-1 (PMC6384874; doi:10.1038/s41598-018-37759-1)
Supplement: Supplementary file 1 — Supplementary Information Photoacoustic transfection of DNA encoding GFP [file 41598_2018_37759_MOESM1_ESM.docx]

**Supplementary Information**

**Photoacoustic transfection of DNA encoding GFP**

Alexandre D. Silva^[[1]](#footnote-1)^, Carlos Serpa^1,^^[[2]](#footnote-2)^* & Luis G. Arnaut^1,*^

**Contents:**

| **Figure S1.** Representative image of the center of the dish with COS-7 cell line labelled with DNA-staining probes (Hoechst 33342 and Propidium Iodide) four hours after exposure to the PA waves. | S3 |
| --- | --- |
| **Figure S2.** Representative image of COS-7 cell line labelled with DNA-staining probes (Hoechst 33342 and Propidium Iodide) four hours after the contact with the metal ring of the PA waves generator device. | S3 |
| **Figure S3**. Representative image of GFP fluorescence by cell line COS-7 24 hours after transfection of the gWizGFP plasmid [100 μg/mL] with 3 minutes exposure to PA waves generated by a 30 ps pulse and 100 mJ/cm^2^ fluence laser. | S4 |
| **Figure S4.** Representative image of GFP fluorescence by cell line COS-7 24 hours after transfection of the gWizGFP plasmid [100 μg/mL] with 5 minutes exposure to PA waves generated by a 30 ps pulse and 100 mJ/cm^2^ fluence laser. | S4 |
| **Figure S5.** Representative image of GFP fluorescence by cell line COS-7 24 hours after transfection of the gWizGFP plasmid [100 μg/mL] with 10 minutes exposure to PA waves generated by a 30 ps pulse and 100 mJ/cm^2^ fluence laser. | S5 |
| **Figure S6.** Representative image of GFP fluorescence by cell line COS-7 24 hours after transfection of the gWizGFP plasmid [100 μg/mL] with 10 minutes exposure to PA waves generated by a 30 ps pulse and 100 mJ/cm^2^ fluence laser. | S5 |
| **Figure S7.** Representative image of GFP fluorescence by cell line COS-7 24 hours after transfection of the gWizGFP plasmid [100 μg/mL] with 10 minutes exposure to PA waves generated by a 30 ps pulse and 100 mJ/cm^2^ fluence laser. | S6 |
| **Figure S8.** Representative image of GFP fluorescence by cell line COS-7 24 hours after transfection of the gWizGFP plasmid [100 μg/mL] with 10 minutes exposure to PA waves generated by a 30 ps pulse and 100 mJ/cm^2^ fluence laser. | S6 |
| **Figure S9.** Representative image of GFP fluorescence by cell line COS-7 24 hours after transfection of the gWizGFP plasmid [100 μg/mL] with 10 minutes exposure to PA waves generated by a 30 ps pulse and 100 mJ/cm^2^ fluence laser. | S7 |
| **Figure S10.** Representative image of GFP fluorescence by cell line COS-7 24 hours after transfection of the gWizGFP plasmid [250 μg/mL] with 10 minutes exposure to PA waves generated by a 30 ps pulse and 100 mJ/cm^2^ fluence laser. | S7 |
| **Figure S11.** Representative image of GFP fluorescence by cell line COS-7 24 hours after transfection of the gWizGFP plasmid [500 μg/mL] with 10 minutes exposure to PA waves generated by a 30 ps pulse and 100 mJ/cm^2^ fluence laser. | S8 |
| **Figure S12.** Representative image of GFP fluorescence by cell line COS-7 24 hours after transfection of the gWizGFP plasmid [100 μg/mL] with 3 minutes exposure to PA waves generated by a 8 ns pulse and 100 mJ/cm^2^ fluence laser. (Left) *Bright field* image; (Centre) Fluorescence microscopy image; (Right) Superposition of the two previous images. | S8 |
| **Figure S13.** Number of transfected cells (average of the three best pictures) 24 hours after transfection of the gWizGFP plasmid [100 μg/mL] with different exposure times to PA waves generated by a 30 ps pulse and 100 mJ/cm^2^ fluence laser. | S9 |
| **Figure S14.** Number of transfected cells (average of the three best pictures) 24 hours after transfection of the gWizGFP plasmid [100 μg/mL] with 10 minutes exposure to PA waves generated by a 30 ps pulse and different laser fluences. | S9 |
| **Figure S15.** Number of transfected cells (average of the three best pictures) 24 hours after transfection of different concentrations of gWizGFP plasmid with 10 minutes exposure to PA waves by a 30 ps pulse and 100 mJ/cm^2^ fluence laser. | S10 |
| **Figure S16.** Number of transfected cells (average of the three best pictures) 24 hours after transfection of the gWizGFP plasmid [100 μg/mL and 250 μg/mL] with 10 minutes exposure to PA waves generated by lasers with 8 ns and 30 ps pulse and 100 mJ/cm^2^ laser fluence. | S10 |


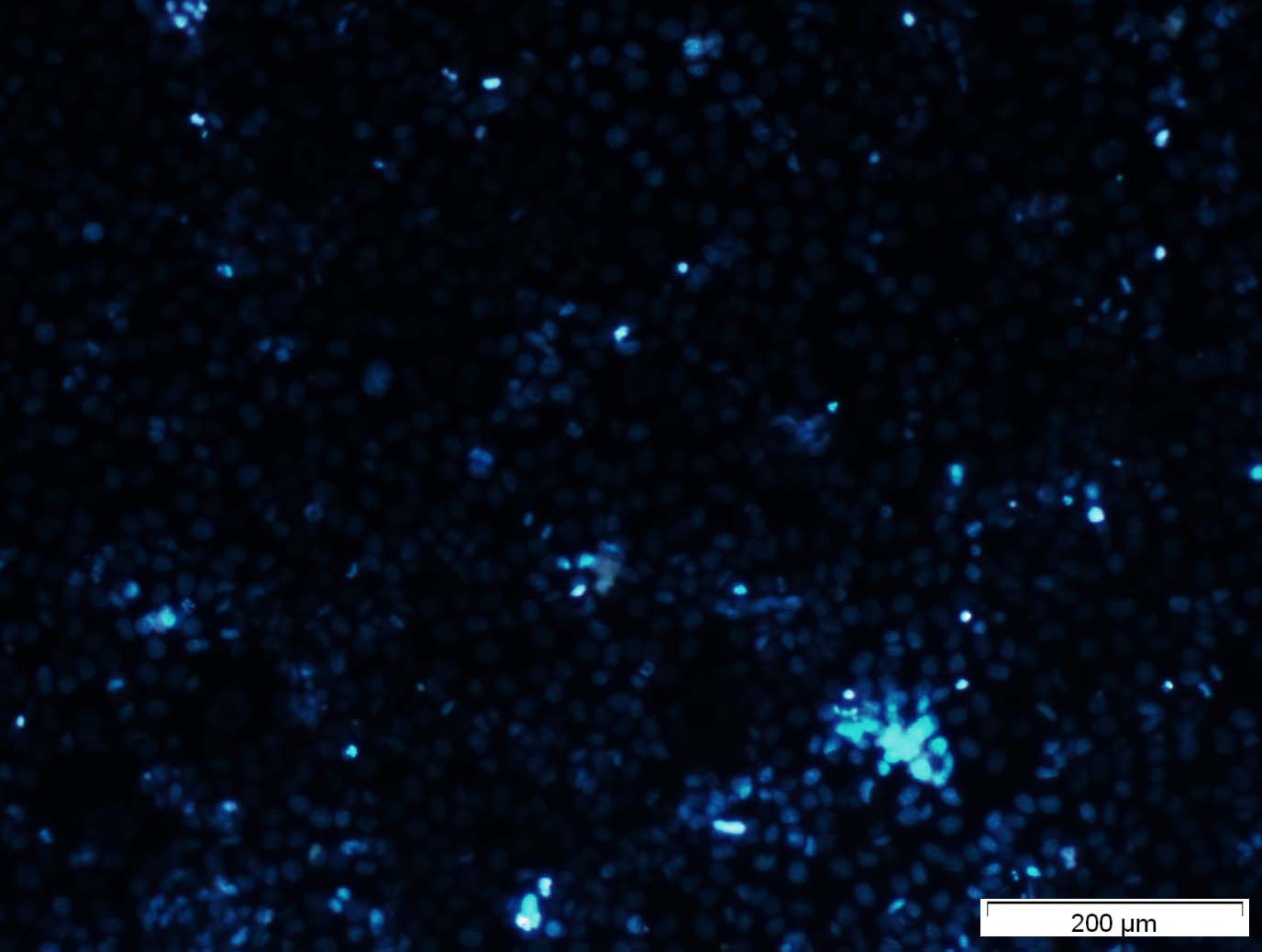


**Figure S1.** Representative image of the center of the dish with COS-7 cell line labelled with DNA-staining probes (Hoechst 33342 and Propidium Iodide) four hours after exposure to the PA waves.


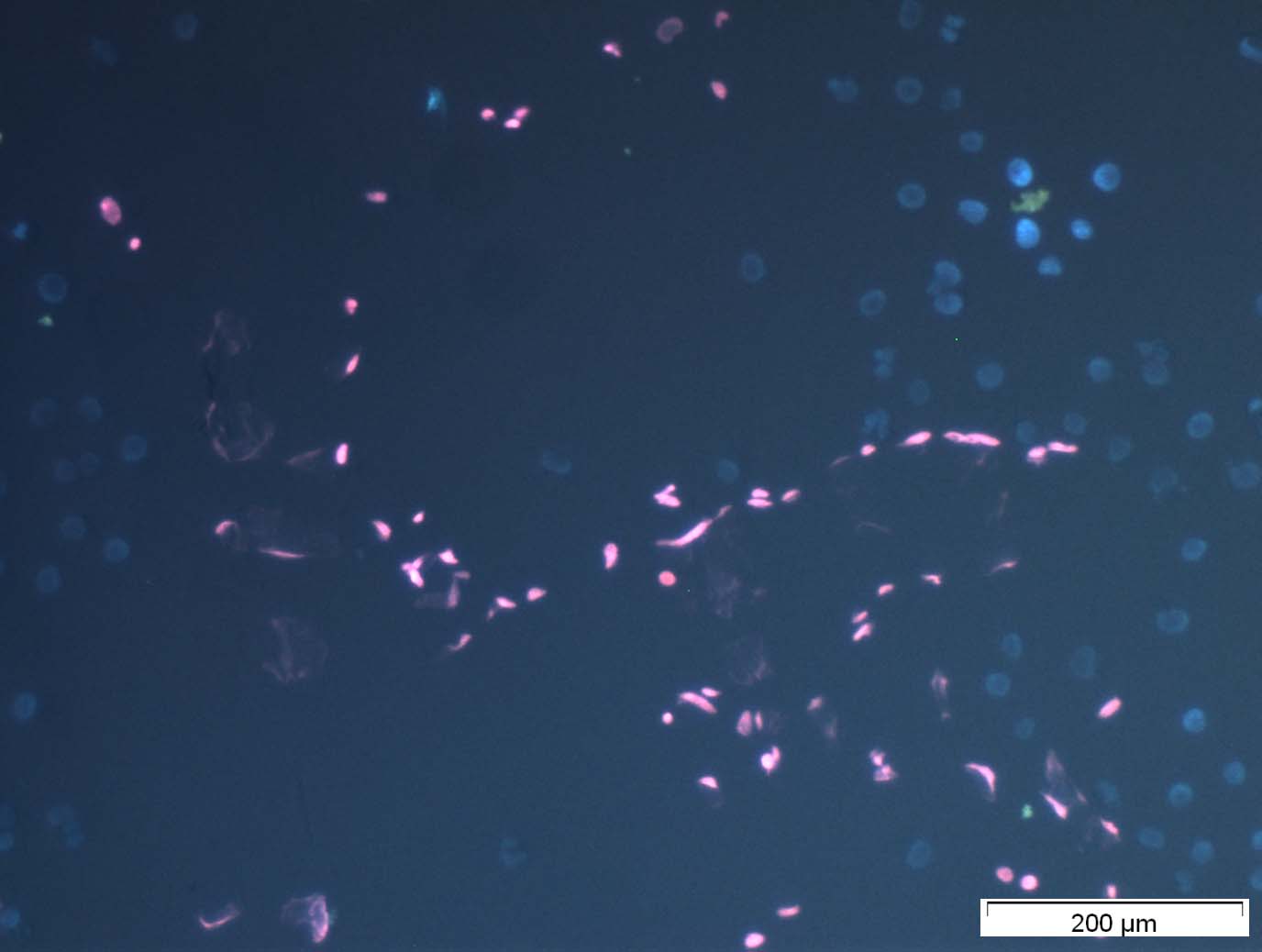


**Figure S2.** Representative image of COS-7 cell line labelled with DNA-staining probes (Hoechst 33342 and Propidium Iodide) four hours after the contact with the metal ring of the PA waves generator device.

**
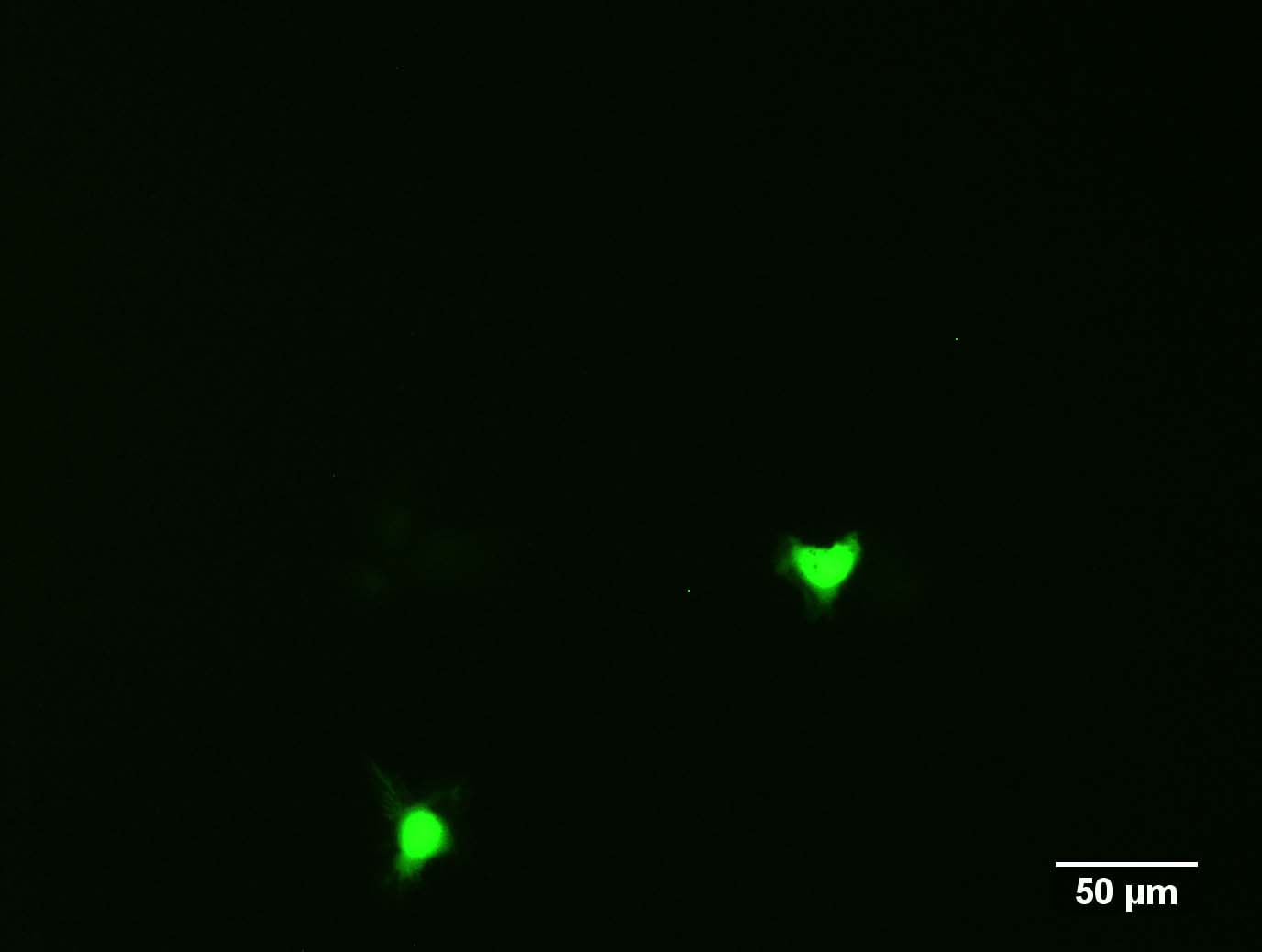
**

**Figure S3**. Representative image of GFP fluorescence by cell line COS-7 24 hours after transfection of the gWizGFP plasmid [100 μg/mL] with 3 minutes exposure to PA waves generated by a 30 ps pulse and 100 mJ/cm^2^ fluence laser.


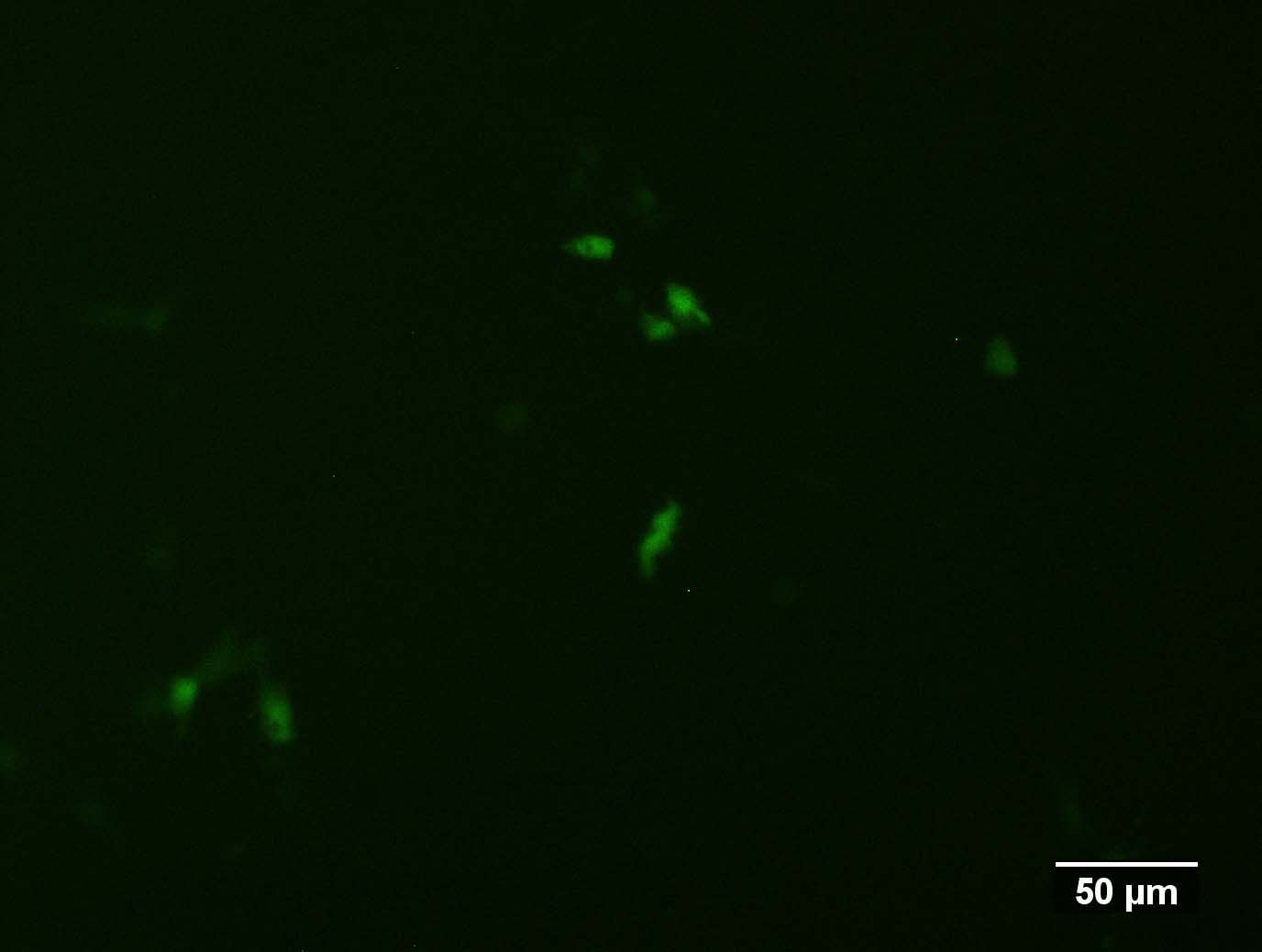


**Figure S4.** Representative image of GFP fluorescence by cell line COS-7 24 hours after transfection of the gWizGFP plasmid [100 μg/mL] with 5 minutes exposure to PA waves generated by a 30 ps pulse and 100 mJ/cm^2^ fluence laser.


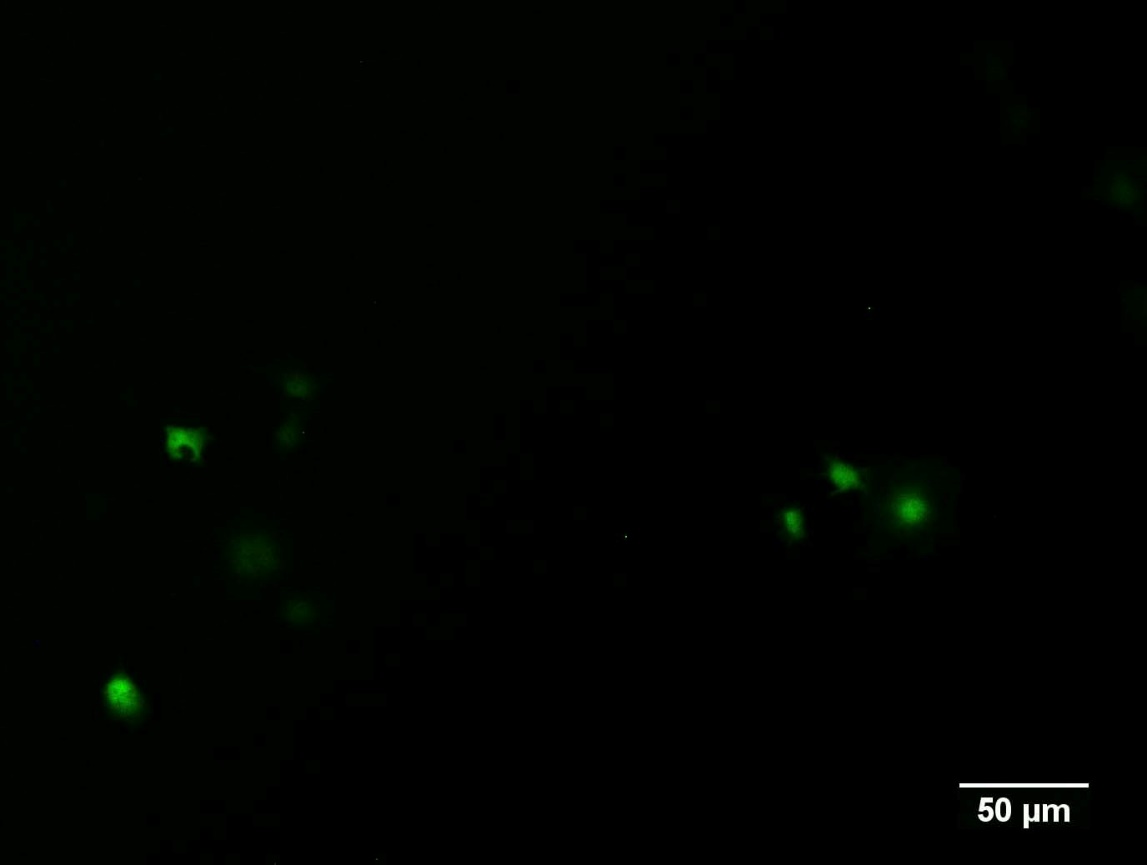


**Figure S5.** Representative image of GFP fluorescence by cell line COS-7 24 hours after transfection of the gWizGFP plasmid [100 μg/mL] with 10 minutes exposure to PA waves generated by a 30 ps pulse and 100 mJ/cm^2^ fluence laser.


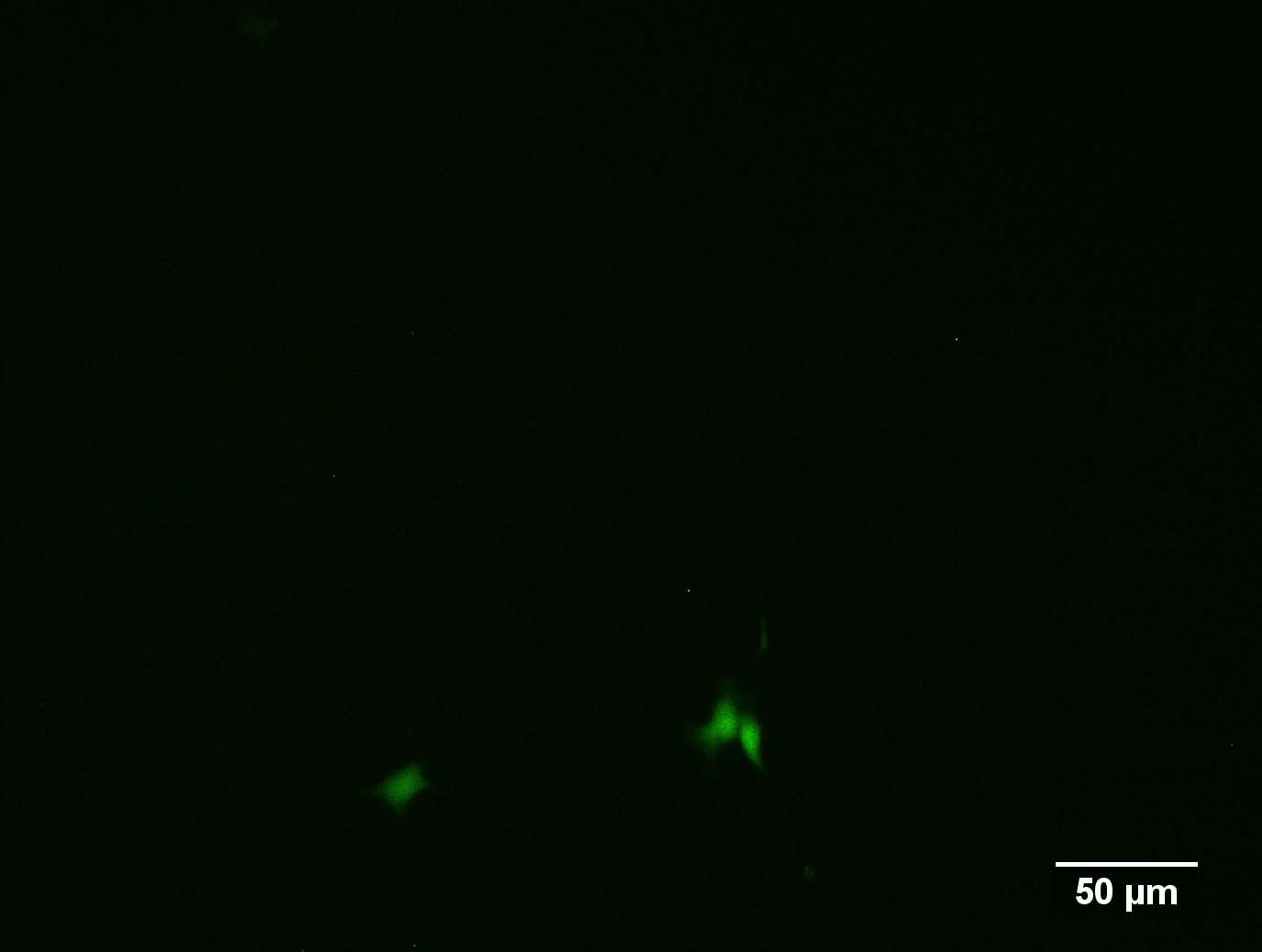


**Figure S6.** Representative image of GFP fluorescence by cell line COS-7 24 hours after transfection of the gWizGFP plasmid [100 μg/mL] with 10 minutes exposure to PA waves generated by a 30 ps pulse and 100 mJ/cm^2^ fluence laser.


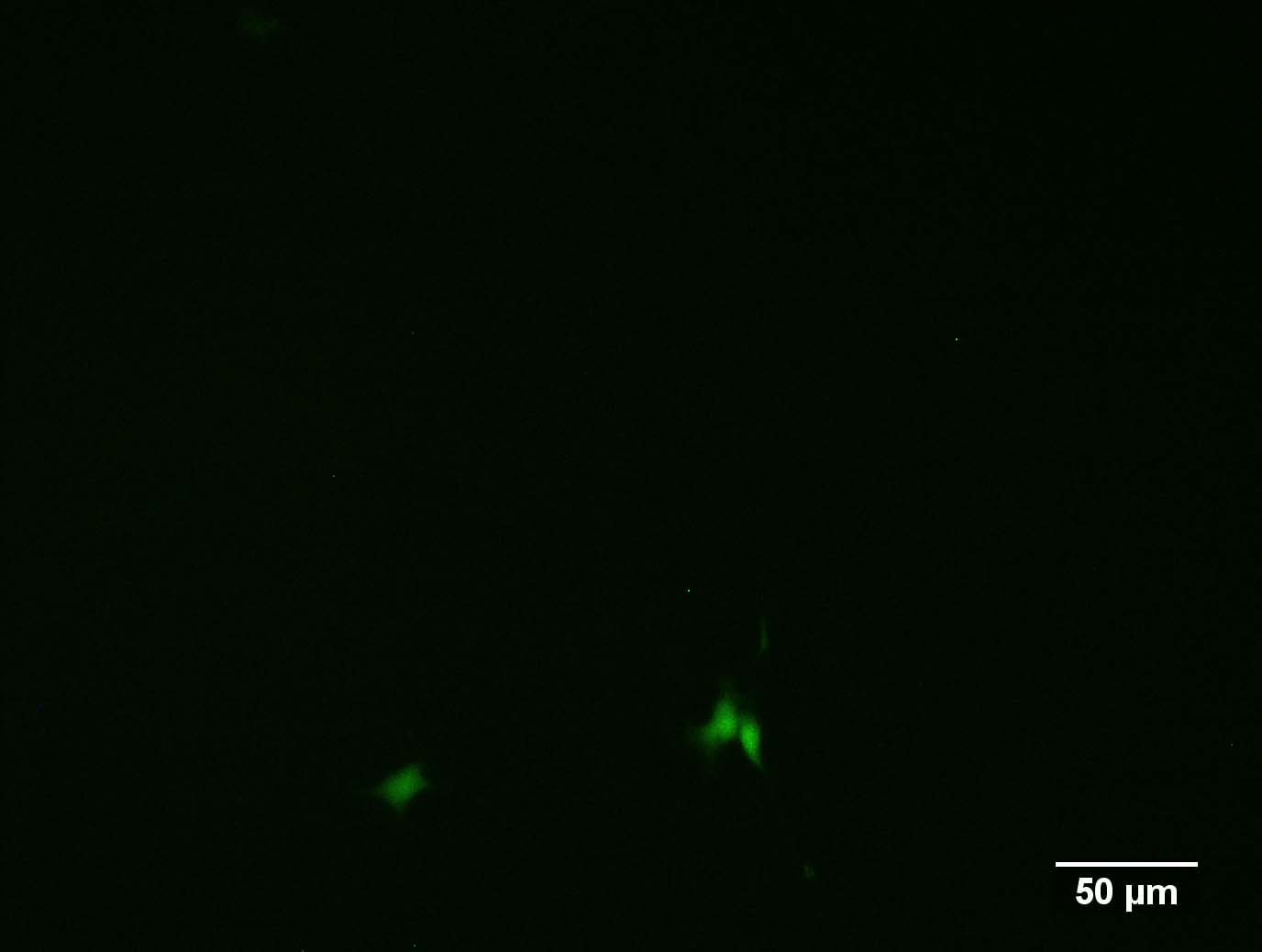


**Figure S7.** Representative image of GFP fluorescence by cell line COS-7 24 hours after transfection of the gWizGFP plasmid [100 μg/mL] with 10 minutes exposure to PA waves generated by a 30 ps pulse and 100 mJ/cm^2^ fluence laser.


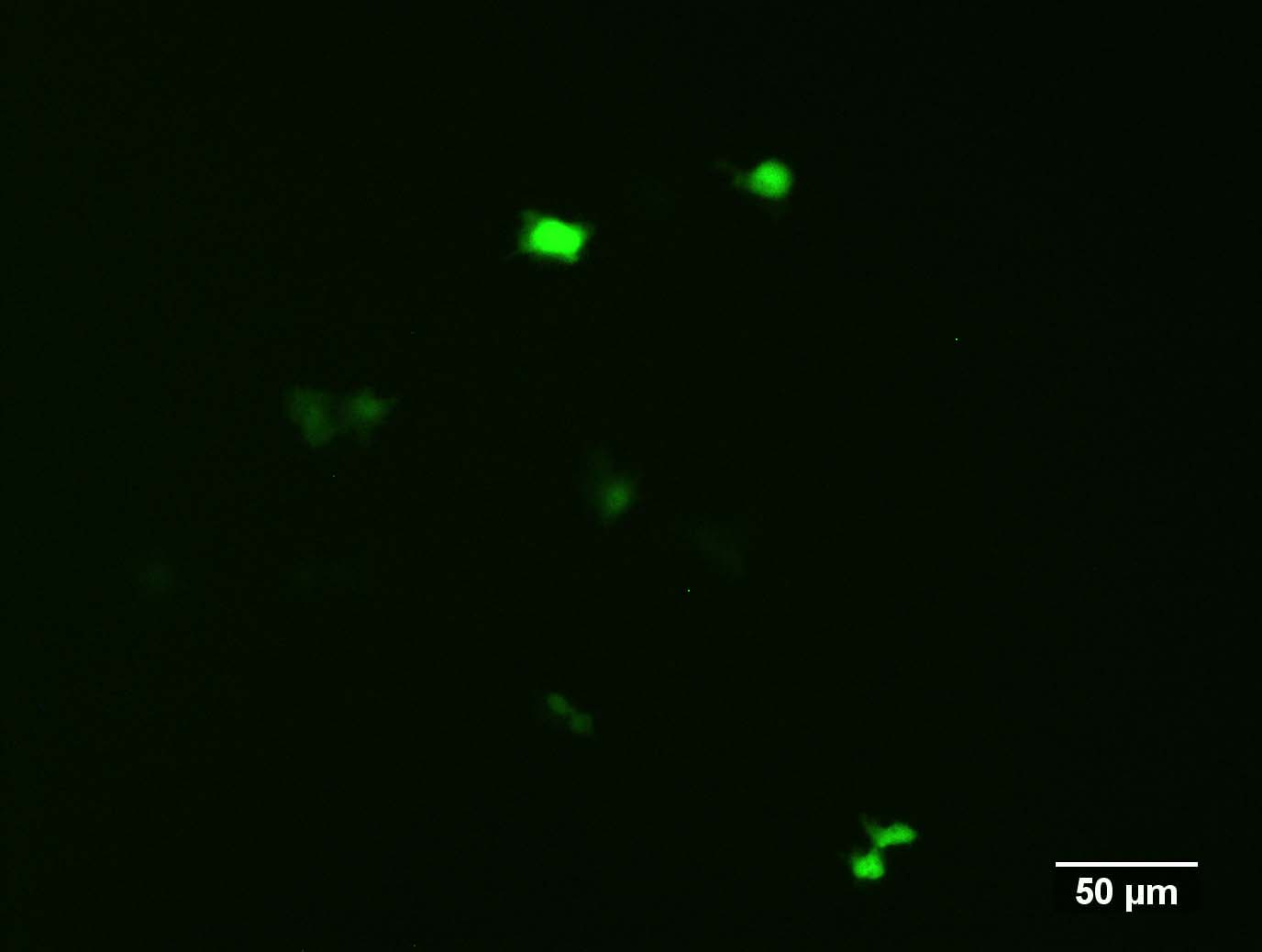


**Figure S8.** Representative image of GFP fluorescence by cell line COS-7 24 hours after transfection of the gWizGFP plasmid [100 μg/mL] with 10 minutes exposure to PA waves generated by a 30 ps pulse and 100 mJ/cm^2^ fluence laser.

**
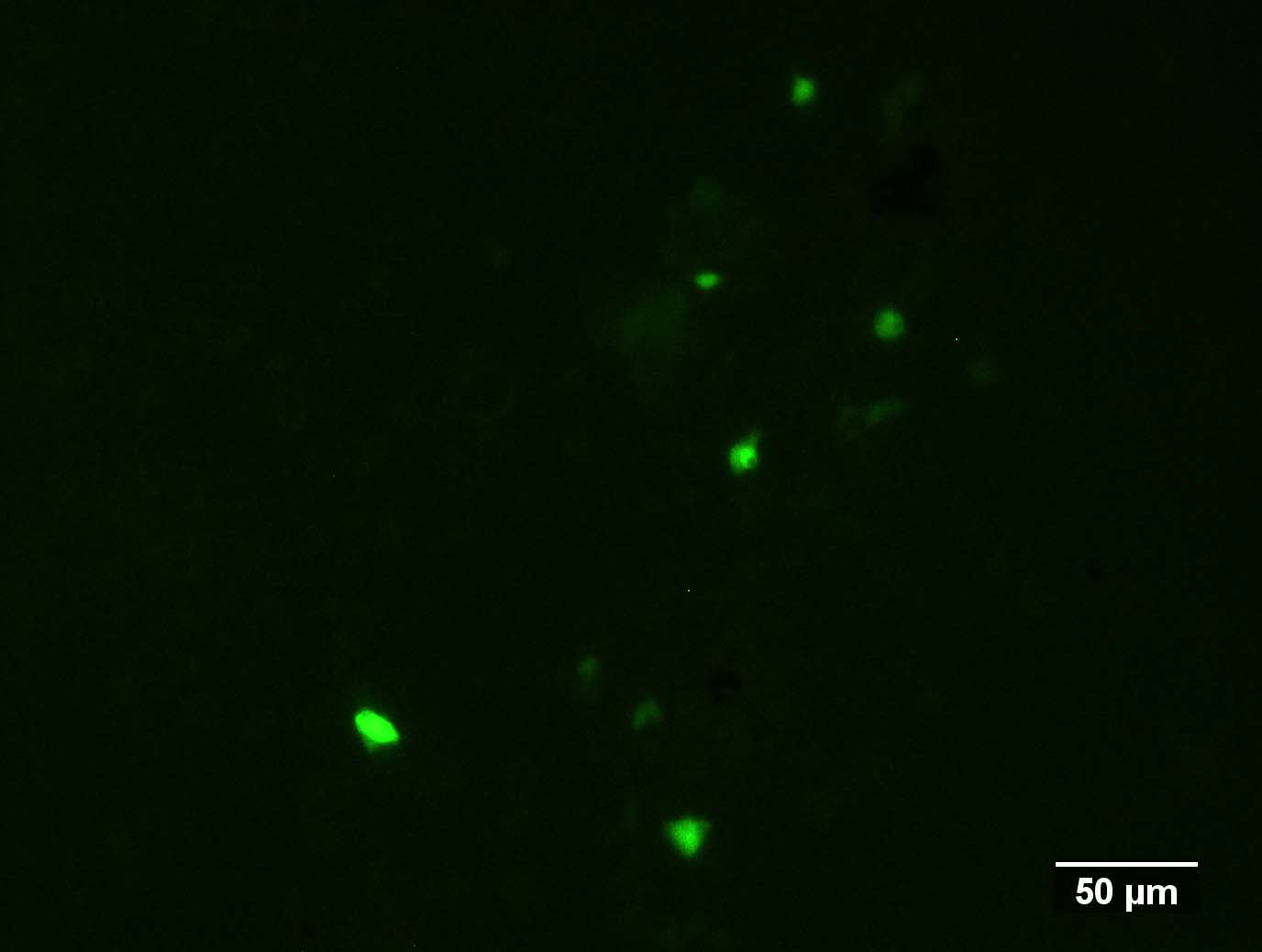
**

**Figure S9.** Representative image of GFP fluorescence by cell line COS-7 24 hours after transfection of the gWizGFP plasmid [100 μg/mL] with 10 minutes exposure to PA waves generated by a 30 ps pulse and 100 mJ/cm^2^ fluence laser.


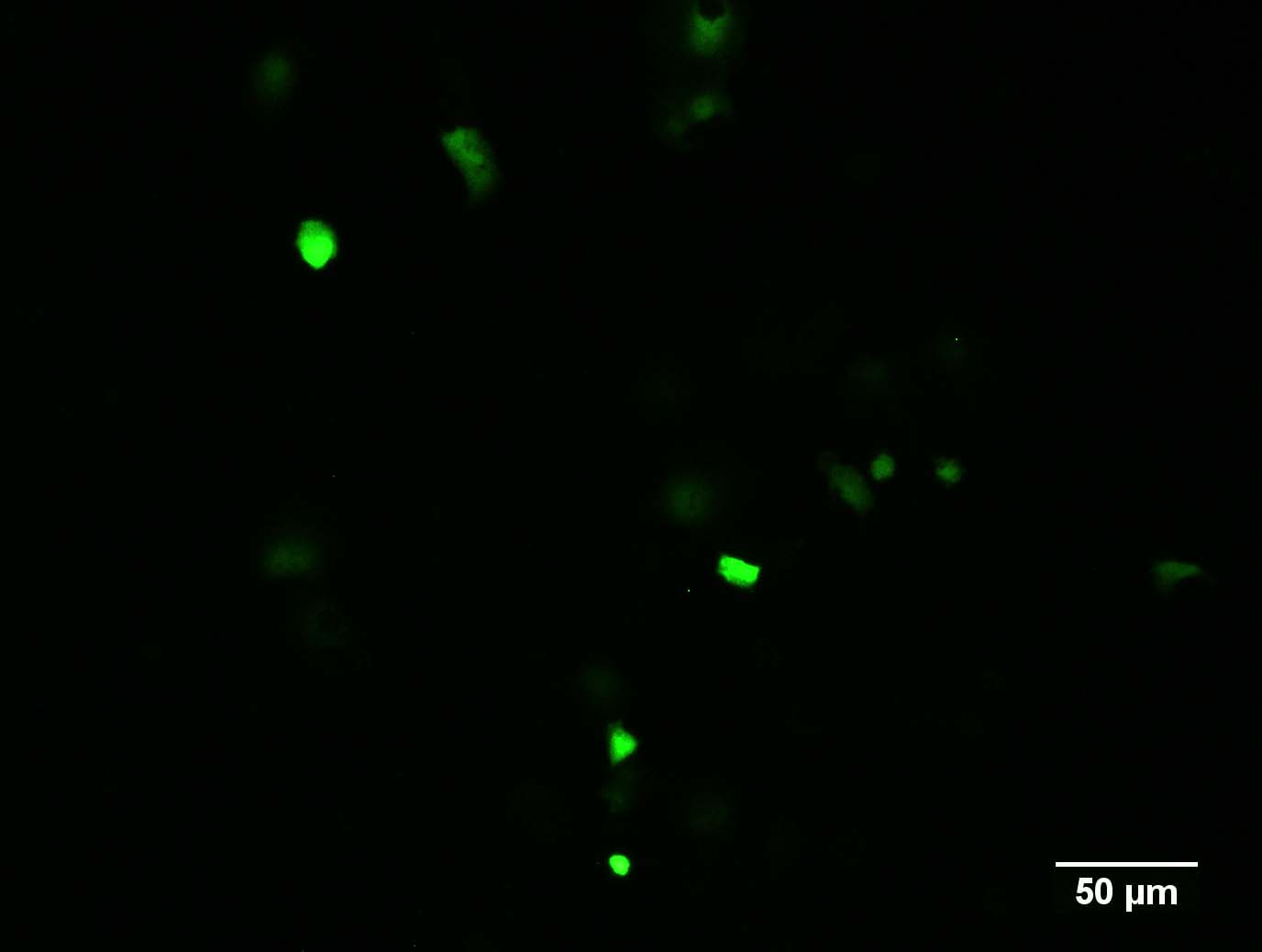


**Figure S10.** Representative image of GFP fluorescence by cell line COS-7 24 hours after transfection of the gWizGFP plasmid [250 μg/mL] with 10 minutes exposure to PA waves generated by a 30 ps pulse and 100 mJ/cm^2^ fluence laser.

**
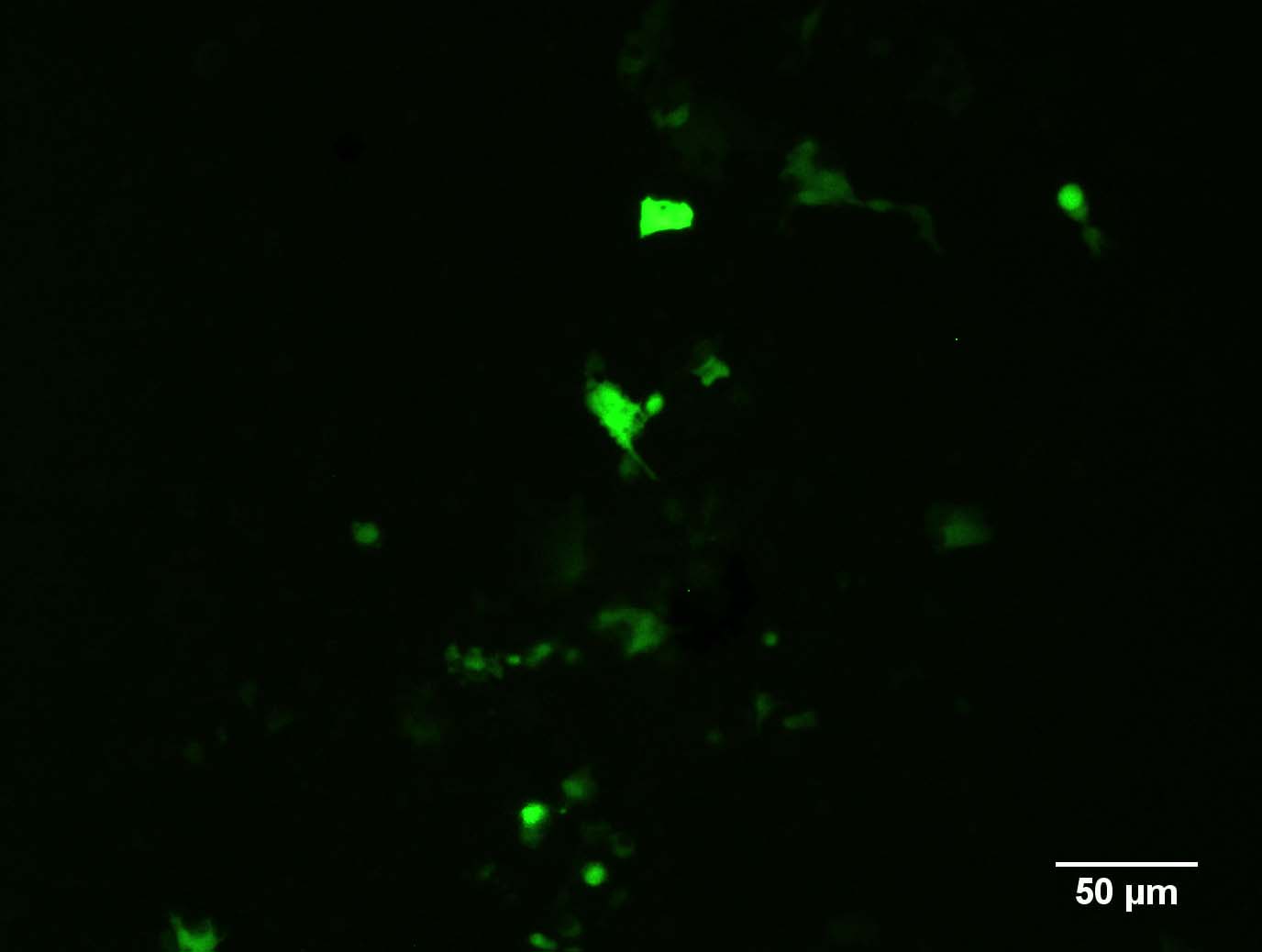
**

**Figure S11.** Representative image of GFP fluorescence by cell line COS-7 24 hours after transfection of the gWizGFP plasmid [500 μg/mL] with 10 minutes exposure to PA waves generated by a 30 ps pulse and 100 mJ/cm^2^ fluence laser.


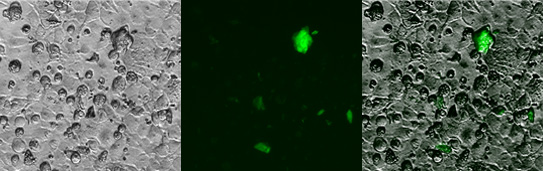


**Figure S12.** Representative image of GFP fluorescence by cell line COS-7 24 hours after transfection of the gWizGFP plasmid [100 μg/mL] with 3 minutes exposure to PA waves generated by a 8 ns pulse and 100 mJ/cm^2^ fluence laser. (Left) *Bright field* image; (Centre) Fluorescence microscopy image; (Right) Superposition of the two previous images.


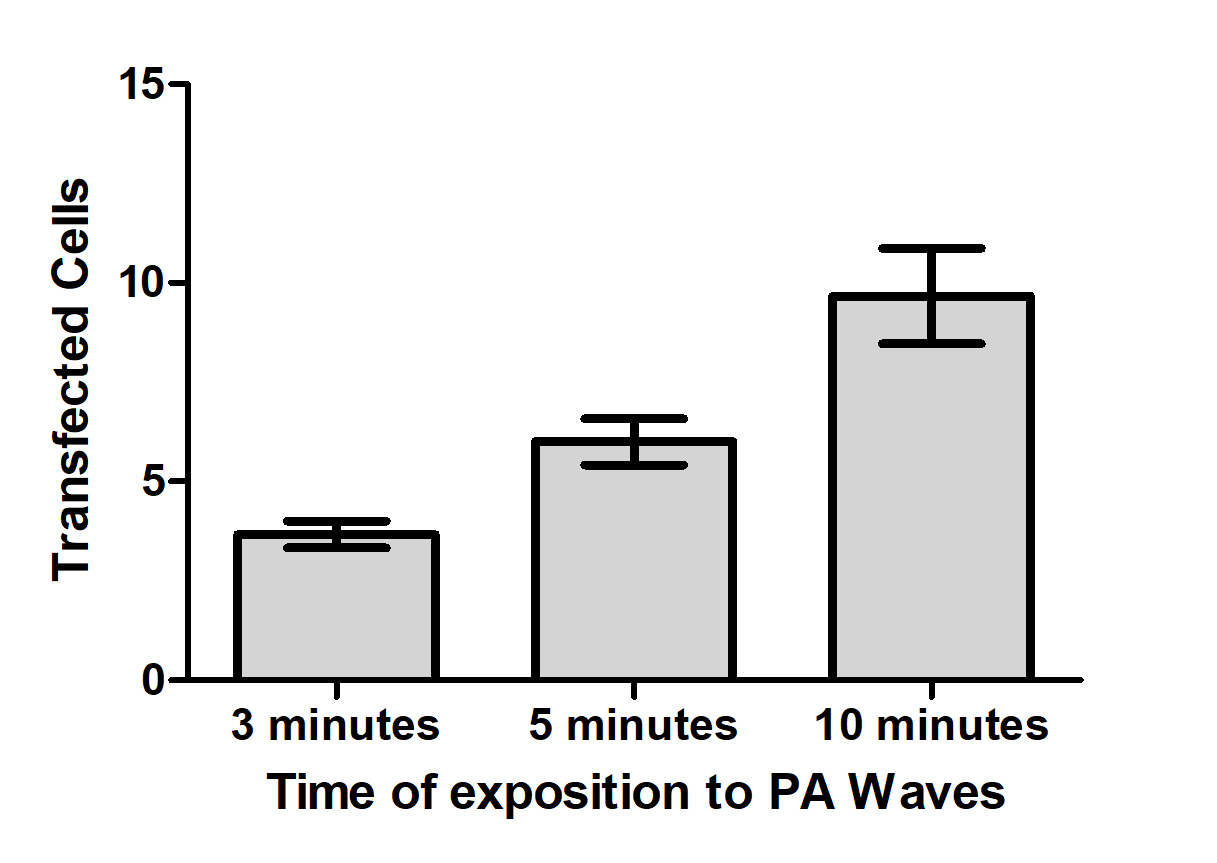


**Figure S13.** Number of transfected cells (average of the three best pictures) 24 hours after transfection of the gWizGFP plasmid [100 μg/mL] with different exposure times to PA waves generated by a 30 ps pulse and 100 mJ/cm^2^ fluence laser.


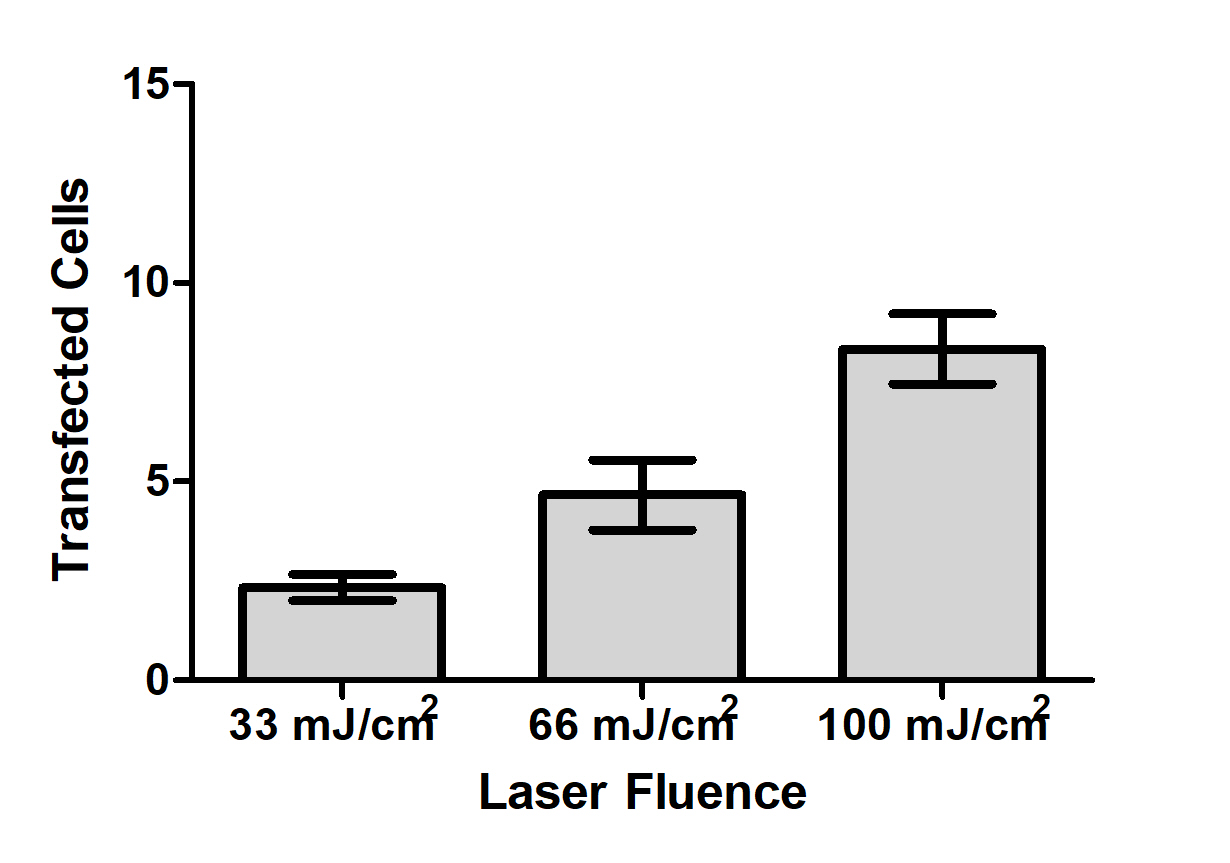


**Figure S14.** Number of transfected cells (average of the three best pictures) 24 hours after transfection of the gWizGFP plasmid [100 μg/mL] with 10 minutes exposure to PA waves generated by a 30 ps pulse and different laser fluences.


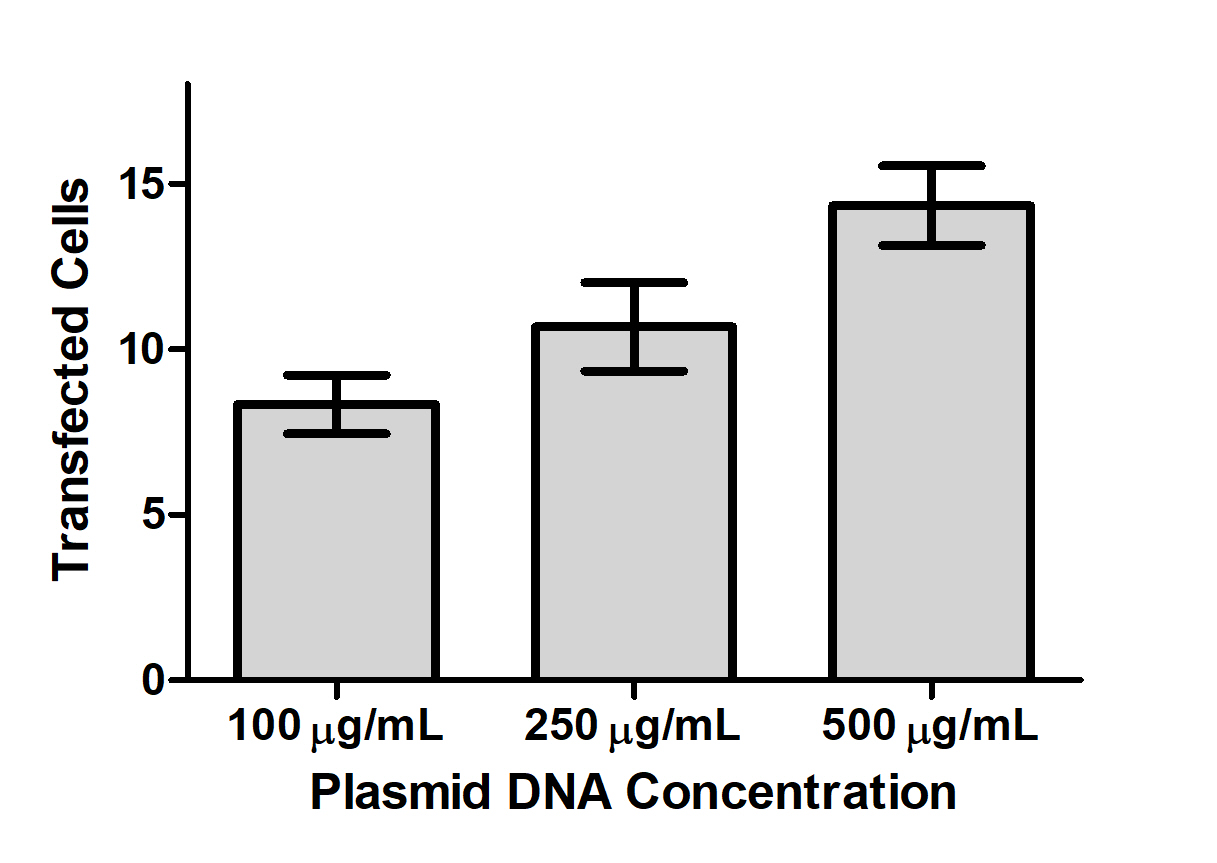


**Figure S15.** Number of transfected cells (average of the three best pictures) 24 hours after transfection of different concentrations of gWizGFP plasmid with 10 minutes exposure to PA waves by a 30 ps pulse and 100 mJ/cm^2^ fluence laser.

**
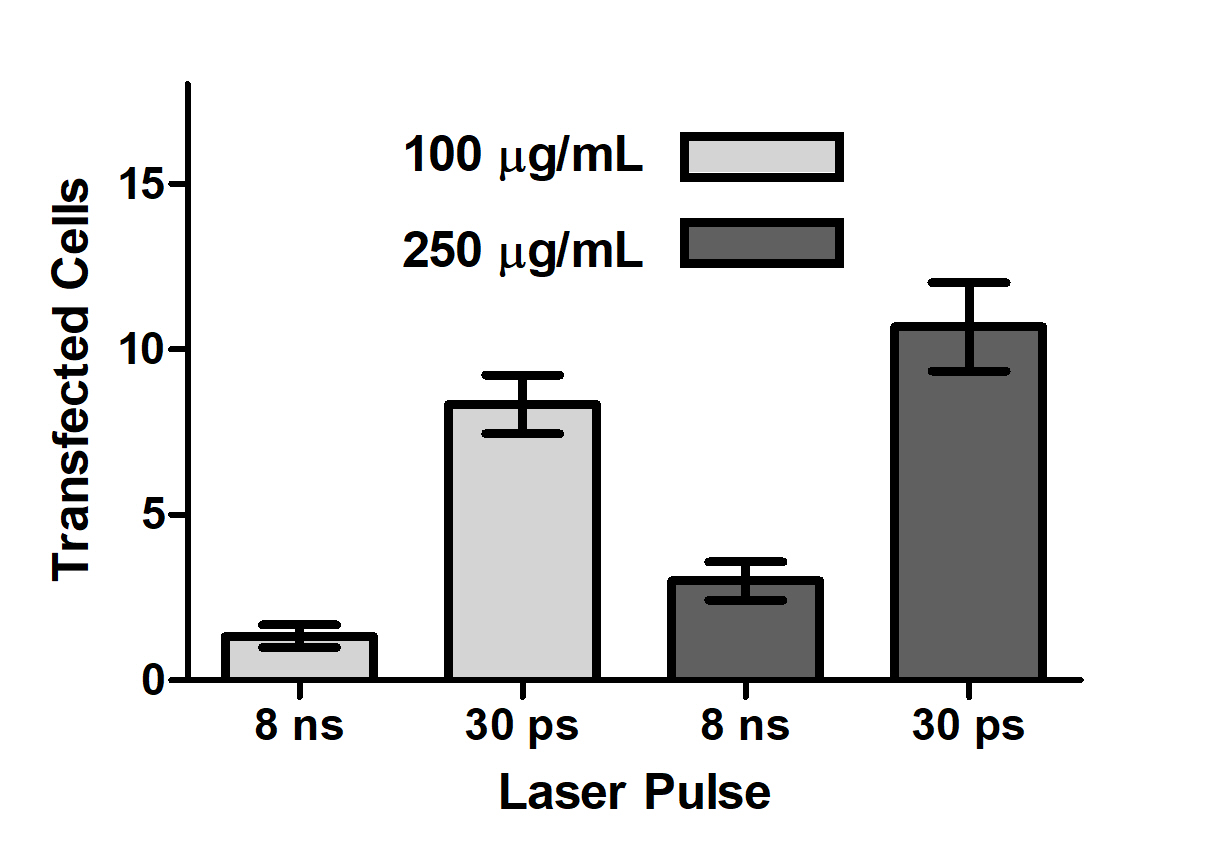
**

**Figure S16.** Number of transfected cells (average of the three best pictures) 24 hours after transfection of the gWizGFP plasmid [100 μg/mL and 250 μg/mL] with 10 minutes exposure to PA waves generated by lasers with 8 ns and 30 ps pulse and 100 mJ/cm^2^ laser fluence.

1. ^1^Department of Chemistry, University of Coimbra, Rua Larga, 3004-535 Coimbra, Portugal [↑](#footnote-ref-1)
2. * Correspondence and requests for materials should be addressed to C.S. (email: [serpasoa@ci.uc.pt](mailto:serpasoa@ci.uc.pt)) or L.G.A. (email: [lgarnaut@ci.uc.pt](mailto:lgarnaut@ci.uc.pt)) [↑](#footnote-ref-2)
